# Photobiomodulation in experimental models of Alzheimer’s disease: state-of-the-art and translational perspectives

**DOI:** 10.1186/s13195-024-01484-x

**Published:** 2024-05-21

**Authors:** Zhihai Huang, Michael R. Hamblin, Quanguang Zhang

**Affiliations:** 1 Department of Neurology, Louisiana State University Health Sciences Center, 1501 Kings Highway, Shreveport, LA 71103 USA; 2 Department of Pharmacology, Toxicology & Neuroscience, Louisiana State University Health Sciences Center, 1501 Kings Highway, Shreveport, LA 71103 USA; 3 Laser Research Centre, University of Johannesburg, Doornfontein, 2028 South Africa

**Keywords:** Alzheimer’s disease (AD), Photobiomodulation (PBM), Neurodegeneration, Therapy, Experimental models

## Abstract

Alzheimer’s disease (AD) poses a significant public health problem, affecting millions of people across the world. Despite decades of research into therapeutic strategies for AD, effective prevention or treatment for this devastating disorder remains elusive. In this review, we discuss the potential of photobiomodulation (PBM) for preventing and alleviating AD-associated pathologies, with a focus on the biological mechanisms underlying this therapy. Future research directions and guidance for clinical practice for this non-invasive and non-pharmacological therapy are also highlighted. The available evidence indicates that different treatment paradigms, including transcranial and systemic PBM, along with the recently proposed remote PBM, all could be promising for AD. PBM exerts diverse biological effects, such as enhancing mitochondrial function, mitigating the neuroinflammation caused by activated glial cells, increasing cerebral perfusion, improving glymphatic drainage, regulating the gut microbiome, boosting myokine production, and modulating the immune system. We suggest that PBM may serve as a powerful therapeutic intervention for AD.

## Introduction

Alzheimer’s disease (AD) is a progressive neurodegenerative disorder affecting millions of people across the world [1]. With the continued aging of the population, the prevalence of AD is expected to further rise in the coming decades [2]. AD is characterized by the extracellular accumulation of amyloid-beta (Aβ) plaques and the presence of intracellular neurofibrillary tangles (NFTs). However the etiology of AD involves a complicated pathophysiology [3], and there is no agreement on the most important causes of AD. Individuals with AD typically experience progressive cognitive impairment, and face challenges in problem-solving, language, and other cognitive functions [3, 4]. Despite an enormous scientific effort focusing on this disease, there are currently limited disease-modifying therapies. To date, several anti-amyloid monoclonal antibodies have been approved for treating early AD patients. Nevertheless, the high cost and potential adverse effects of these medications have driven scientists to explore alternative therapeutic hypotheses and strategies [5–8]. In this context, treatment strategies for this disease have been proposed to shift from single to multiple targets, and a vast array of new therapeutic approaches for AD is under investigation [9, 10].

Photobiomodulation (PBM), also known as low-level laser (light) therapy, is a promising modality to treat a wide variety of pathological conditions, such as wound healing, pain, inflammation, and tissue injury [11, 12]. PBM is a non-pharmacological and non-invasive intervention, involving exposing cells or tissue to low levels of red and/or near-infrared (NIR) light (wavelengths between 600–1100 nm) [13]. This low-level irradiation triggers various biological processes, including the modulation of mitochondrial dynamics, inhibition of inflammatory and apoptotic signaling, as well as the secretion of neurotrophic factors [14, 15]. Recently, the application of PBM therapy for neurological conditions, particularly AD and other age-related neurodegenerative diseases, has sparked increasing interest [12, 16]. The therapeutic potential of PBM for AD or dementia has been widely reported in experimental animal models and in some preliminary clinical trials [17–20].

In this review, we summarize the current understanding of the biological mechanisms underlying the therapeutic benefits of PBM for AD, with a focus on knowledge gained from studies in preclinical animal models. We then discuss the limitations of existing studies, as well as the challenges inherent in clinical translation. We hope this state-of-the-art review will provide a guide for future research in this area.

## Treatment paradigms of PBM for AD

The dose of PBM for therapeutic purposes can be determined by dividing the total irradiation time (in seconds) by the power output (in mW/cm^2^), then dividing the result by 1000. This value is expressed as J/cm^2^ and is known as the fluence or energy density. Several different paradigms for treating brain diseases with PBM have been reported. These include transcranial, intranasal, intravascular, and systemic PBM, as well as the recently proposed remote PBM [12, 21]. Given the limited research on intranasal and intravascular PBM approaches, this review will primarily focus on the biological mechanisms of transcranial, systemic, and remote PBM.

Transcranial PBM is the most studied PBM paradigm for the treatment of AD. This involves the non-invasive delivery of visible and/or NIR light, typically using light-emitting diodes (LED) applied to the head and brain regions for appropriate lengths of time (typically 10—30 min) [13, 22]. The primary effects of transcranial PBM with various light parameters in cell, animal, and human studies have been systematically reviewed elsewhere [23, 24]. These reviews highlight the potential of this therapy against neurodegeneration. In contrast, systemic PBM tested in preclinical studies has involved placing animals under an irradiation apparatus and exposing their entire bodies to an LED array located overhead [25, 26]. Various whole-body light pods have also been developed to offer a more accessible PBM treatment option for human patients [27, 28].

Remote PBM is a novel concept involving the application of PBM to peripheral tissues/organs such as the abdomen or the legs, which has been suggested to indirectly affect the brain via as yet unknown mediators [21, 29]. This approach stems from the observation that PBM can elicit systemic effects that contribute to the protection of distant tissues [30]. In an analogy to the technique of remote ischemic conditioning, scientists have coined the term “remote PBM” to describe this phenomenon [30]. Despite the limited understanding of its mechanisms to date, experimental animal studies have reported some neuroprotective effects of remote PBM against AD. Therefore remote PBM is considered to be promising for future clinical applications [31–33].

**Fig. 1:**
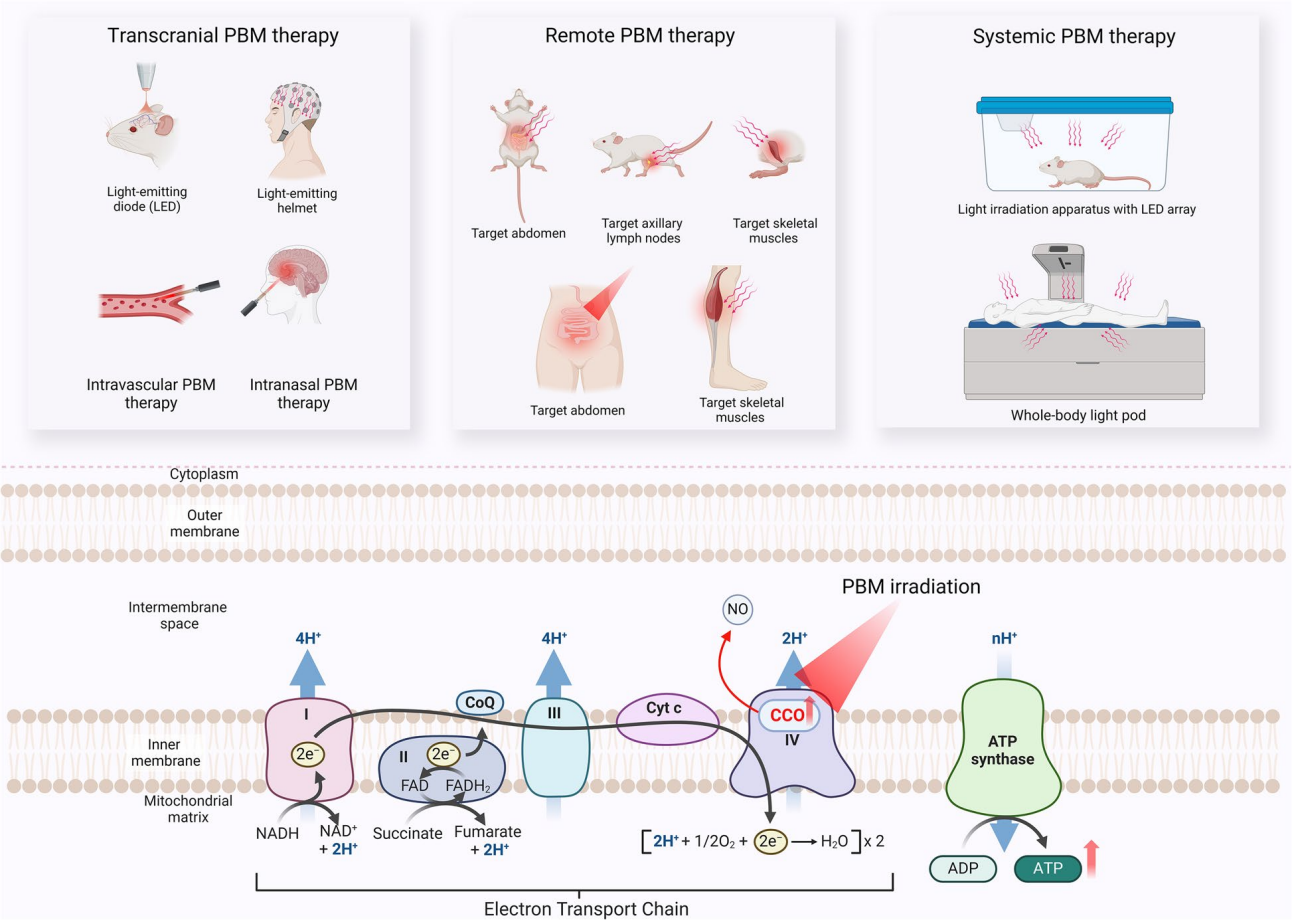
Overview of different PBM treatment paradigms for AD and an illustration showing the mechanisms traditionally considered to underlie the action of PBM. Abbreviations: LED, light-emitting diode; PBM, photobiomodulation; CCO, cytochrome c oxidase; ATP, adenosine triphosphate; NO, nitric oxide

Regardless of the specific treatment paradigm employed, the well-accepted mechanism underlying the effects of PBM is that the light can be absorbed by cytochrome c oxidase (CCO), the terminal enzyme of the mitochondrial electron transport chain (ETC) [34, 35]. This enzyme catalyzes the transfer of electrons from cytochrome c to molecular oxygen in the terminal step of the ETC. This electron transfer process is coupled with the pumping of protons across the inner mitochondrial membrane, establishing an electrochemical gradient that drives the synthesis of adenosine triphosphate (ATP) by ATP synthase [36]. CCO contains two heme centers and two copper centers, which serve as molecular chromophores to absorb photons of red-to-near-infrared wavelengths [22, 37]. The absorption of photons delivered by PBM increases the activity of CCO and the synthesis of ATP, boosting mitochondrial function and triggering the initiation of various cellular processes [22, 38]. PBM may also lead to the dissociation of inhibitory nitric oxide (NO) from the CCO molecule, which could further facilitate electron transfer [39]. However, recent evidence suggests that CCO may not be the only target of PBM. In cells lacking CCO, PBM can still exert biological effects [40]. Additionally, various new biological effects of PBM have been reported recently, which will be discussed in detail in the following chapters. A diagram illustrating PBM treatment paradigms and the general mechanisms of action is provided in Fig. 1.

## Biological mechanisms underlying the benefits of transcranial PBM for AD

### PBM-activated cellular signaling

Historically, the accumulation of Aβ plaques and NFTs formed by pathologically assembled tau proteins has been considered central to the progression of AD. Aβ plaques and NFTs are thought to be the primary contributors, initiating diverse signaling cascades that ultimately result in synaptic dysfunction and neural degeneration [41, 42]. Intriguingly, mounting evidence suggests that transcranial PBM could attenuate these AD pathological hallmarks and signaling cascades. A 4-week treatment with transcranial PBM (670 nm, continuous wave, 4 J/cm^2^ applied to the head daily) has been reported to reduce hyperphosphorylated tau and neurofibrillary tangles in K3 mice, a transgenic mouse model with tau pathology [43]. Using the same parameters, a reduced Aβ burden was also observed in APP/PS1 mice, a transgenic mouse model engineered to develop Aβ pathology [43].

**Fig. 2:**
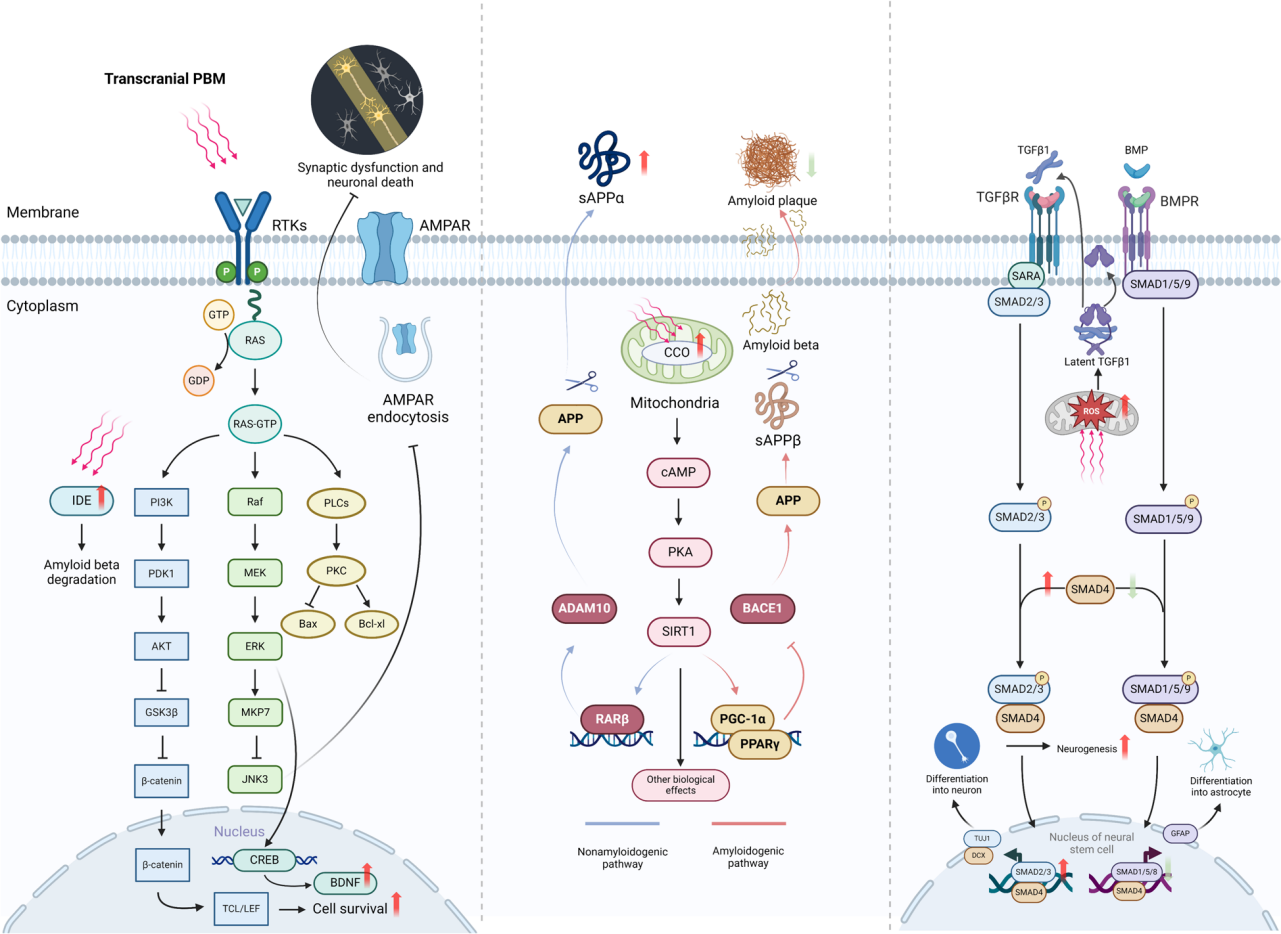
Summary of signaling pathways activated by transcranial PBM and subsequent biological effects. Abbreviations: PBM, photobiomodulation; RTKs, receptor tyrosine kinases; AMPAR, α-amino-3-hydroxy-5-methyl-4-isoxazolepropionic acid receptor; IDE, insulin-degrading enzyme; CREB, cAMP response element-binding protein; BNDF, brain-derived neurotrophic factor; CCO, cytochrome c oxidase; APP; amyloid precursor protein; PGC1-α, peroxisome proliferator-activated receptor gamma coactivator 1-alpha; PPARγ, peroxisome proliferator-activated receptor gamma; ADAM10, a disintegrin and metalloproteinase domain-containing protein 10; TGFβ-1, transforming growth factor beta 1, TGFβR, transforming growth factor beta receptor; BMP, bone morphogenetic protein; BMPR; Bone morphogenetic protein receptor; ROS, reactive oxygen species

Research during the past decade has significantly advanced our understanding of the cellular signaling pathways activated by transcranial PBM (Fig. 2). In cultured neuron-like cells, PBM treatment (632.8 nm, continuous wave, 0.156 J/cm^2^-0.624 J/cm^2^, single treatment) triggered PKC signaling-dependent upregulation of the anti-apoptotic protein Bcl-2 and inhibited the pro-apoptotic protein Bax, thereby preventing Aβ^25−35^-induced cellular apoptosis [44].

Other in vitro studies have shown that PBM can target the Akt/GSK3b/β-catenin pathway to counteract Aβ-induced cell apoptosis [45]. PBM treatment (632.8 nm, continuous wave, 2 J/cm^2^, single treatment) activated Akt while also inactivating the GSK3b/β-catenin pathway. This, in turn, led to the enhanced nuclear translocation of β-catenin and increased its TCF/LEF-dependent transcriptional activity, thus promoting cell survival [45].

Transcranial PBM could also alter the signaling pathways implicated in Aβ processing. Aβ peptide is derived from the cleavage of amyloid precursor protein (APP). The processing of APP involves two distinct pathways; the non-amyloidogenic pathway and the amyloidogenic pathway [46, 47]. In the amyloidogenic pathway, APP is cleaved by β-secretase, also known as β-site APP cleaving enzyme 1 (BACE1). This cleavage generates soluble APPβ (sAPPβ). The remaining C-terminal fragment subsequently undergoes proteolytic processing by γ-secretase, resulting in the production of Aβ peptides [48]. Conversely, in the non-amyloidogenic pathway, APP is cleaved by α-secretase, e.g. a disintegrin and metalloproteinase domain-containing protein 10 (ADAM10) [49]. This cleavage yields soluble APP alpha (sAPPα) and a C-terminal fragment, precluding the formation of Aβ peptides [49]. Therefore, approaches that lead to the switching of APP processing into the non-amyloidogenic pathway have been proposed to be a disease-modifying strategy for AD [48, 50]. Of note, in a transgenic AD mouse model, a-30 day transcranial PBM treatment (632.8 nm, continuous wave, 2 J/cm^2^ at the hippocampus level daily) shifted APP processing toward the nonamyloidogenic pathway [51]. APP/PS1 mice treated with transcranial PBM showed a reduced Aβ burden, while the levels of full‐length APP and the main proteolytic enzymes (insulin-degrading enzyme, IDE and neprilysin, NEP) responsible for Aβ degradation remained unchanged [51]. Consistent with these findings, transcranial PBM also increased the level of sAPPα and decreased the level of sAPPβ, accompanied by the upregulation of ADAM10 and downregulation of BACE1 protein levels. In vitro experiments further suggested that this shift in the APP processing pathway was mediated by increased CCO activity and subsequent activation of the PKA/SIRT1 signaling pathway [51]. Interestingly, long-term transcranial PBM appears to elicit more profound biological effects than short-term treatment. Indeed, a decrease in sirtuin1, encoded by the SIRT1 gene, has been linked to the progression of AD [52–54]. A postmortem study has revealed that reduced brain sirtuin1 aligns with the accumulation of tau in AD patients [55]. As a key metabolic regulator, SIRT1 exhibits neuroprotective properties, such as inhibiting tau hyperphosphorylation, alleviating oxidative stress and neuroinflammation, and modulating synaptic plasticity [56]. These aspects have been extensively discussed in previous reviews [53, 56]. Notably, a recent study demonstrated that PBM therapy restored SIRT1 expression in chronically stressed mice [57]. This suggests that PBM might activate SIRT1/sirtuin1 and associated signals, therefore exerting diverse biological effects. Additionally, despite the exact mechanism remaining unknown, a 14-week transcranial PBM treatment (610 nm, continuous wave, 2 J/cm^2^ daily), initiated at 2 months of age, significantly elevated the levels of IDE, with reduced Aβ accumulation and less neuronal loss [58].

Transcranial PBM could also protect against AD-associated synaptic dysfunction by attenuating AMPA receptor endocytosis [59]. Synaptic dysfunction is another major pathological process that might be responsible for memory impairment in the progression of AD [60, 61]. AMPA receptors, typically located on the postsynaptic membrane of neurons, mediate fast excitatory synaptic transmission [62]. When activated by neurotransmitters, AMPA receptors are internalized into the postsynaptic neuron, a process known as AMPA receptor endocytosis [62, 63]. The dynamic interplay between AMPA receptor endocytosis and exocytosis, leading to the insertion of receptors into the membrane, allows synapses to adapt to changes in neuronal activity. This process facilitates synaptic plasticity, the basic foundation of learning and memory [64, 65]. Aberrant AMPA receptor endocytosis has been implicated in AD-associated memory impairment [66, 67]. Moreover, the inhibition of AMPAR endocytosis could prolong memory retention in normal animals and ameliorate memory impairment in an experimental animal model of AD [68]. Reduction of Aβ^1−42^ results in JNK signaling-dependent endocytosis of surface AMPA receptors and ameliorates subsequent dendrite injury [59]. In contrast, transcranial PBM treatment (632.8 nm, continuous wave, 2 J/cm^2^ at the hippocampus level daily for 30 days) in APP/PS1 mice activated the ERK/MKP7 signaling pathway, which further inhibited JNK3 to attenuate AMPA receptor endocytosis and rescued synaptic pathology [59].

As a non-pharmacological intervention, transcranial PBM recently has been proven to modulate neurogenesis, and trigger the formation of new neurons from neural stem and progenitor cells [69, 70]. The dentate gyrus of the hippocampus serves as a central hub for adult neurogenesis in mammals [71]. While there is a growing body of evidence suggesting that hippocampal neurogenesis likely continues throughout life, it has been shown to diminish with aging and is compromised in AD [71, 72]. Targeted stimulation of neurogenesis has been shown to restore AD-linked impairment of synaptic formation and memory in an experimental animal model [73]. Notably, the fate of neural stem cells is regulated by various signaling pathways, including TGFβ and BMP signaling, which govern their differentiation into either neurons or astrocytes [74, 75]. In one recent study using both in vivo and in vitro models, it was observed that PBM (in vivo, 635 nm, continuous wave, 6 J/cm^2^ at cortex level daily, for 1 month; in vitro, 2 J/cm^2^, single treatment) boosted the interaction of the transcription factors Smad2/3 with Smad4 and triggered the differentiation of neural stem cells into immature neurons, which resulted in enhanced neurogenesis [70]. Moreover, photoactivation of Smad2/3 competitively reduced the interaction of Smad1/5/9 with Smad4, leading to decreased differentiation into astrocytes [70]. This shift in the direction of neural stem cell differentiation is mediated by the production of reactive oxygen species (ROS) in response to PBM. This, in turn, activated the TGFβ/Smad signaling pathway, initiating downstream effector molecules and cellular processes [70]. PBM (632.8 nm, continuous wave, 0.5, 1, 2, and 4 J/cm^2^, single treatment) could also activate the transcription factor CRE-binding protein (CREB) in an ERK-dependent manner [76]. This activation of CREB further triggered the expression of brain-derived neurotrophic factor (BDNF), a neurotrophin implicated in neuronal survival, differentiation, and plasticity [76, 77]. The inhibition of ERK abrogated PBM-activated CREB/BDNF, thereby eliminating the beneficial effects of PBM in mitigating Aβ-induced neuron loss and dendritic atrophy [76].

Transcranial PBM may also affect intraneuronal signaling. In cultured primary neurons, PBM treatment mitigated neuronal damage induced by oxygen and glucose deprivation, leading to the restoration of neuronal viability [78]. Nevertheless, the potential effects of transcranial PBM on intraneuronal signaling in the context of AD remain to be demonstrated by future research.

Taken together, transcranial PBM appears capable of activating a variety of intracellular signaling molecules and signaling pathways. In light of the multifactorial nature of AD, the ability of transcranial PBM to initiate multiple intracellular signals with different beneficial effects encourages optimism concerning its therapeutic potential in AD.

### PBM and mitochondrial function

One important biological effect of PBM is its ability to regulate mitochondrial function [38, 39]. PBM can increase mitochondrial membrane potential, partially through regulating mitochondrial redox signaling, thus leading to increased ATP production [39]. Intriguingly, under normal physiological conditions, PBM appears to increase intracellular ROS and trigger downstream signaling pathways. Conversely, under pathological conditions with already existing oxidative stress, PBM tends to inhibit ROS production by restoring the mitochondrial potential to its healthy state [39]. Utilizing 31P magnetic resonance spectroscopy, it was found that 670 nm transcranial PBM (20 min) increased the ATP synthase flux rate in the brains of older adults [79]. This observation was initial clinical evidence supporting the efficacy of PBM in enhancing mitochondrial function in the human brain.

**Fig. 3:**
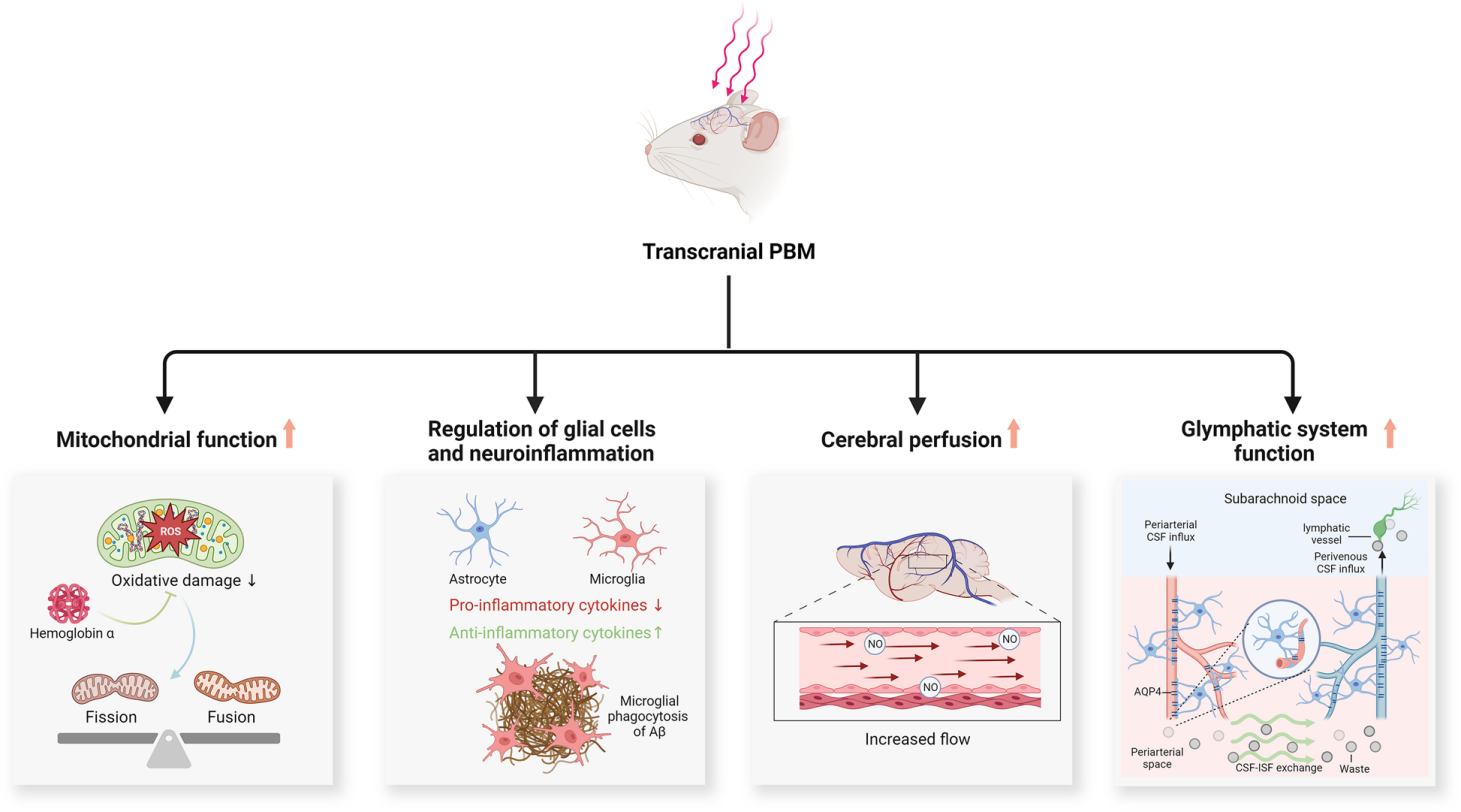
Additional proposed mechanisms of action for transcranial PBM. Abbreviations: PBM, photobiomodulation; ROS, reactive oxygen species; Aβ, amyloid beta; NO, nitrogen oxide; CSF, cerebrospinal fluid; AQP4, aquaporin 4; ISF, interstitial fluid

Emerging research suggests that mitochondrial dysfunction, which may either derive from Aβ deposition or occur independently of Aβ pathology, potentially presents a brain “energy crisis” and contributes to the onset of AD [80, 81]. Two recent studies have presented evidence that transcranial PBM could rescue mitochondrial dysfunction observed in the progression of AD [17, 82]. A 5-day transcranial PBM (808 nm, continuous wave, 3 J/cm^2^ at the cortex level and ~ 1 J/cm^2^ at the hippocampus level daily) rescued Aβ^1−42^ infusion-triggered mitochondrial dysfunction [82]. This intervention conferred multiple benefits, including the preservation of mitochondrial dynamics, inhibition of mitochondrial fragmentation, restoration of mitochondrial membrane potential, facilitation of mitochondrial homeostasis, and increased cytochrome c oxidase activity and ATP synthesis [82]. In another study, a transgenic rat model of AD was used to investigate the efficacy of long-term transcranial PBM preconditioning for AD [17]. At 18 months of age, TgF344-AD rats exhibited increased mitochondrial fragmentation, accompanied by oxidative damage and abnormal mitochondrial dynamics, characterized by an increase in mitochondrial fission proteins and a reduction in mitochondrial fusion proteins [17]. Strikingly, daily transcranial PBM treatment (from 2 to 18 months old, 3 times/week, 808 nm, continuous wave, 3 J/cm^2^ at the cortex level) reversed these pathological alterations and restored mitochondrial homeostasis [17]. Further experiments revealed that the beneficial effects conferred by PBM were mediated by neuronal hemoglobin α, a component of nerve cells essential for intraneuronal oxygen homeostasis [17, 83]. A diagram illustrating these findings is presented in Fig. 3.

### PBM, glial cells, and neuroinflammation

Studies in recent years have firmly established neuroinflammation to be a central player in AD pathogenesis [84, 85]. Astrocytes and microglia, two major neural cell types, are recognized as key contributors to the initiation of the inflammatory response in the brain [86, 87]. In response to injury, disease, or infection in the CNS, activated microglia and astrocytes release both inflammatory mediators and neuroprotective factors [88, 89]. These factors may play distinct roles in shaping the local microenvironment [90, 91]. Despite the limited understanding of these cellular processes and the complexity of the physiology of glial cells, their “double-edged sword” role in AD has been recognized [92, 93].

Preliminary findings suggest that transcranial PBM may modulate the polarization of glial cells to limit pro-inflammatory signals [17, 82, 94]. Microglial cells exposed to Aβ exhibit increased levels of pro-inflammatory cytokines, which could be suppressed by PBM treatment (808 nm, continuous wave, 9 J/cm^2^, single treatment) [94]. Furthermore, there is evidence that transcranial PBM triggers the polarization of microglia and astrocytes into neuroprotective/anti-inflammatory phenotypes [17]. The TgF344-AD rat model shows elevated pro-inflammatory M1 microglia and A1 pro-inflammatory astrocytes in the brain. Similar results were observed in microglial and astrocytal cultures treated with Aβ [17]. Remarkably, both in vivo transcranial and in vitro treatment with PBM (808 nm, continuous wave, 3 J/cm^2^ at the cortex level, three times per week, last for 16 months) shifted pro-inflammatory glial cells into anti-inflammatory/neuroprotective phenotypes. This transformation was accompanied by decreased levels of pro-inflammatory cytokines and elevated levels of anti-inflammatory cytokines [17]. As the resident immune cells of the CNS, microglia are implicated in the clearance of Aβ through phagocytosis, a physiological process negatively correlated with inflammatory mediators [95, 96]. Application of 808 nm PBM in this context, appears to activate microglia-mediated phagocytosis of Aβ. This was shown by the increased recruitment of microglia to the surrounding amyloid plaques following PBM treatment [17, 94]. Another study revealed the role of microglia-derived exosomes in the anti-inflammatory effects conferred by PBM [97]. In cultured BV-2 microglial cells, PBM (1070 nm, 10 Hz, 2 and 4 J cm^2^) remarkably inhibited Aβ-triggered inflammation, an effect that was abolished by the blockade of exosome biogenesis/release [97]. Moreover, the administration of exosomes derived from microglia exposed to 1070-nm light attenuated neuroinflammation and improved spatial learning and memory ability in 5xFAD AD mice [97]. This suggests that the contents of microglia-derived exosomes are crucial for the anti-inflammatory effects of PBM. These findings offer new mechanistic insights into the regulatory effects of PBM on inflammation and glial cells.

### PBM and cerebral perfusion

Normal cerebral perfusion is crucial for maintaining proper neuronal function. Disruptions in perfusion can negatively impact both brain structure and function, potentially contributing to cognitive decline in the elderly population [98, 99]. Moreover, global and regional cerebral hypoperfusion has been widely observed in AD throughout its course [100–102]. A recent cross-sectional study also suggested an elevated risk of mild cognitive impairment and dementia associated with cerebral hypoperfusion [103].

Although direct research on the impact of transcranial PBM on AD-associated cerebral hypoperfusion is presently lacking, the available evidence suggests that PBM may play a role in sustaining regional cerebral blood flow [19]. In a pilot study, fourteen patients with mild cognitive impairment underwent transcranial PBM (applied to the vertebral and internal carotid arteries), and regional perfusion was measured at baseline and after PBM treatment [19]. While no statistically significant differences were observed post-treatment, notable trends emerged in certain brain regions, including the medial prefrontal cortex, lateral prefrontal cortex, anterior cingulate cortex, and occipital lateral cortex [19]. Furthermore, these patients reported enhanced overall cognitive function after PBM treatment [19]. PBM irradiation over the right prefrontal cortex also enhanced regional cerebrovascular oxygenation in healthy adults [104]. One animal study offered mechanistic insights into the regulation of cerebral perfusion in response to transcranial PBM [105]. Transcranial PBM (808 nm, 1.6 W/cm^2^ for 15–45 min) increased local CBF by 30% compared to control animals, accompanied by an elevation in cerebral NO, a crucial factor in cerebral blood flow regulation [105, 106]. Importantly, the effect of PBM could be abolished by administering L-NAME, an inhibitor of NO synthase [105].

It should be noted that the impaired bioavailability of NO has been proposed to be a mechanism underlying cognitive decline and AD-associated cerebral hypoperfusion [107]. A study using cultured human endothelial cells and human neuroblastoma cells provided further insight into the mechanisms behind PBM-induced NO production [108]. Three different PBM parameters (808 nm, 1064 nm, and 1270 nm, continuous wave, 10 mW/cm^2^) triggered the release of NO from human endothelial cells, accompanied by an elevated expression of phosphorylated endothelial nitric oxide synthase (eNOS) [108]. In contrast, cultured neuronal cells expressing neuronal nitric oxide synthase (nNOS) showed no appreciable NO generation following PBM irradiation, suggesting that increased eNOS and its phosphorylation may play a pivotal role in PBM-triggered NO production. A recent study further uncovered that phosphorylation of eNOS is crucial for enhanced CBF following transcranial PBM treatment [109]. A 5-min exposure to a 1064-nm laser at an irradiance of 50 mW/cm2 significantly elevated CBF levels compared to baseline, accompanied by an increase in eNOS phosphorylation. Conversely, mice incapable of phosphorylating eNOS exhibited no change in CBF after the same PBM treatment procedure [109].

Although remaining controversial, it has been proposed that the dissociation of inhibitory NO from CCO by the action of PBM may also contribute to increased NO levels [39]. Consequently, the enhanced NO levels resulting from PBM may increase cerebral blood flow, therefore counteracting AD-associated cerebral hypoperfusion. The proposed mechanism for this effect is outlined in Fig. 3.

### PBM and the glymphatic system

The glymphatic system, also known as the meningeal lymphatic system, is a recently discovered macroscopic waste clearance system in the brain [110]. The glymphatic system comprises perivascular spaces, astroglial cells, interstitial spaces in neural tissue, and perivenous spaces. It plays a crucial role in the clearance of soluble proteins (e.g., Aβ) and other metabolites from the CNS [110, 111]. In the glymphatic system, cerebrospinal fluid (CSF) penetrates the brain through the periarterial spaces. Subsequently, CSF traverses into the interstitium via the interaction of perivascular astrocytic and aquaporin-4 (AQP4), facilitating the drainage of interstitial fluid (ISF) and its solutes through perivenous pathways [112, 113]. Furthermore, AQP4, a key member of the aquaporin water channel protein family, is prominently expressed on the end feet of astrocytes near the vascular perivascular spaces. This polarized distribution of AQP4 is thought to facilitate the rapid exchange of CSF with ISF and support effective glymphatic drainage [114, 115].

An impaired function of the glymphatic system is a common feature of various neurodegenerative disorders, including AD, Parkinson’s disease, and multiple sclerosis [116–118]. Compromised AQP4 polarization and the limited exchange of CSF and ISF have been observed in an experimental animal model of AD [115, 116]. Inhibition or deletion of AQP4 potentiated the deposition of phosphorylated tau in the brain, along with the exacerbation of neurodegeneration [115, 116]. Similarly, suppressing glymphatic drainage by ligating deep cervical lymph nodes aggravated Aβ plaque formation and memory impairment in 5xFAD mice [119]. A recent neuroimaging study demonstrated a negative correlation between whole-brain glymphatic activity and the deposition of amyloid and tau, along with positive correlations with cognitive scores [120].

Notably, several lines of evidence suggest that transcranial PBM may serve as a non-invasive method to enhance glymphatic drainage and clearing functions [121–123]. When treated with 1267 nm transcranial PBM (continuous wave, 4 J/cm^2^ for pups and 9 J/cm^2^ for adult mice at the cortex level, for 7 days), there was a significant improvement in the removal of infused macromolecules from the lateral ventricle into deep cervical lymph nodes [121]. This effect was achieved through the dilation of basal meningeal lymphatic vessels triggered by PBM [121]. Similarly, employing these PBM parameters also enhanced the clearance of macromolecules through the glymphatic pathway in mice infused with Aβ and promoted the aggregation of Aβ around meningeal lymphatic vessels [122, 123]. Notably, PBM typically involves modulating cellular responses through non-thermal mechanisms. The wavelength range, starting from 1100 nm and above is thought to be absorbed by water, potentially causing heating effects and damage to brain tissues [124]. However, measurements at the the surface of cortex indicated that transcranial PBM at 1267 nm, did not induce any alterations in brain temperature and no morphological changes were observed [121]. This suggests the potential application of transcranial PBM at longer wavelengths.

A more recent study has provided direct evidence of transcranial PBM’s effect on AD-associated glymphatic pathology [125]. In 6-month-old 5xFAD AD mice, a 4-week transcranial PBM intervention (continuous wave, 6–30 J/cm^2^) significantly alleviated learning and memory deficits while enhancing glymphatic drainage [125]. Notably, further investigation revealed that the enhanced lymphatic drainage largely mediates the beneficial effects of PBM, as the ablation of meningeal lymphatic vessels abolishes PBM’s effect on cognitive improvement. Additionally, mice treated with PBM exhibited enhancements in mitochondrial metabolism and cellular junctions of meningeal lymphatic endothelial cells, potentially underlying the positive effects of PBM on glymphatic drainage [125]. Taken together, these findings may point to new mechanistic pathways underlying the beneficial effects of transcranial PBM for AD.

## Biological mechanisms underlying the benefits of remote PBM for AD

### Abdomen-targeted PBM

Over the past few decades, the close communication between the gut microbiome and CNS functions has been increasingly recognized, leading to a concept known as the gut-brain axis [126]. Recent evidence suggests that the gut microbiome may be an important player in the progression of AD [127]. Significantly different gut microbiome compositions have been observed between AD patients and healthy individuals [128, 129]. More importantly, in transgenic rodent models of AD, the transplantation of fecal microbiota from healthy wild-type mice resulted in a marked reduction in amyloid burden and less tau pathology. Conversely, transplantation of microbiota from AD mice induced cognitive impairment and impaired neurogenesis in wild-type mice [130, 131]. Although the precise mechanisms through which the gut microbiome influences brain function remain elusive, the gut microbiome is increasingly regarded as an attractive target for the prevention and management of AD.

It is also intriguing to note that abdominal PBM irradiation may lead to a change in the composition of the gut microbiome. A 2-week daily abdominal irradiation with PBM (660 nm and 808 nm, continuous wave, total fluence 10 J/cm^2^) resulted in a significant difference in intestinal flora diversity compared with control animals. Furthermore, in this pilot study, *Allobaculum*, a genus of bacteria that is associated with a healthy microbiome, was significantly increased following PBM irradiation [132]. In a case report, a human participant received PBM treatment (904 nm; pulsed wave, 700 Hz pulse frequency, 861.3 total joules) on his abdomen for 11 weeks, and his microbiome was tested six times before and after treatment. The results showed substantial changes in intestinal flora diversity following PBM treatment, with an increased abundance of beneficial bacteria such as *Akkermansia*, *Faecalibacterium*, and *Roseburia*, and a decreased abundance of potentially pathogenic genera [133]. While this case study provides valuable insight, it should be noted that this is only one case without any comparison. Also, microbiomes can vary significantly among individuals. Thus, these results have to be viewed with caution. To validate these results, further case–control studies with larger participant cohorts are imperative.

**Fig. 4:**
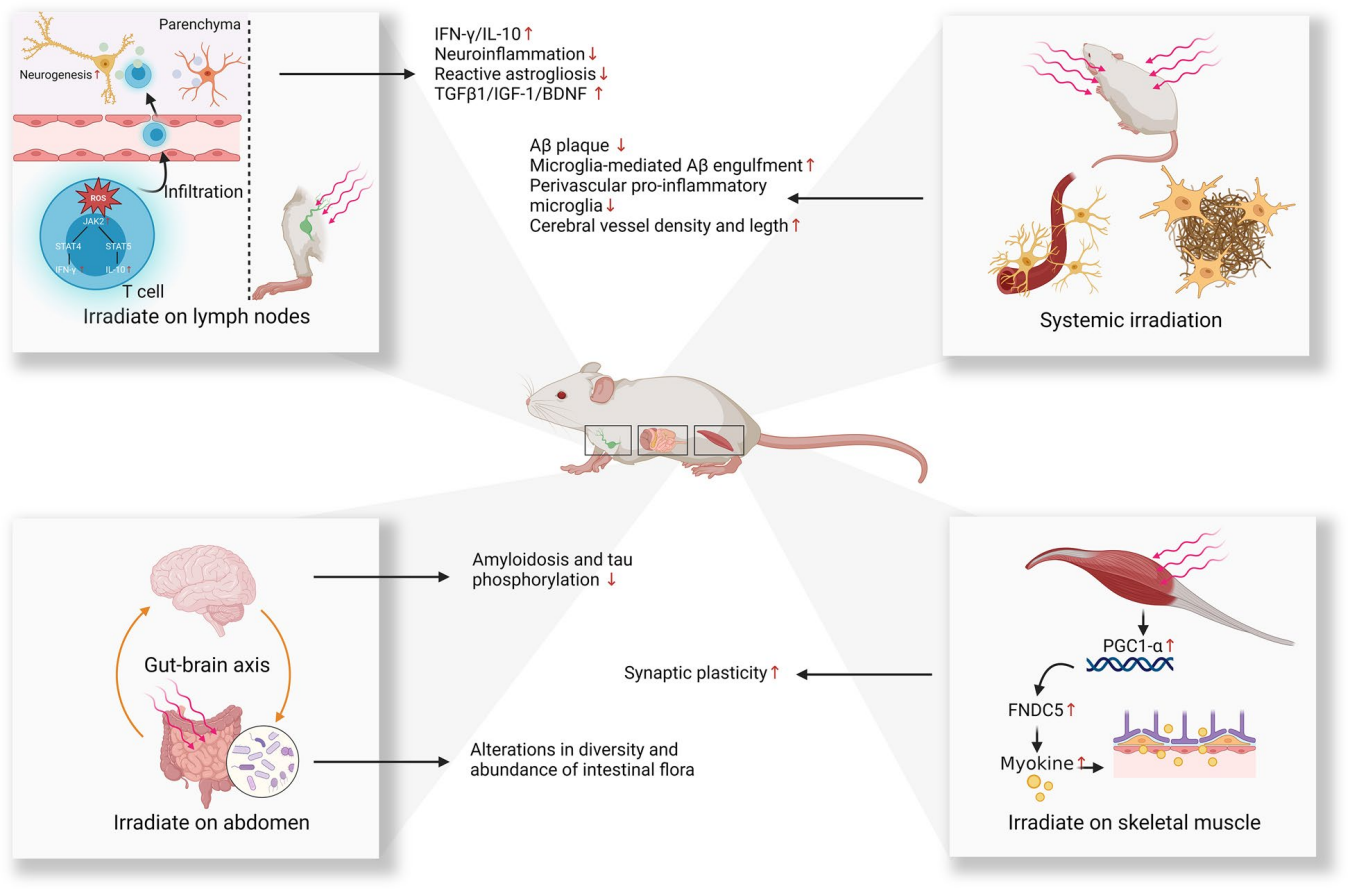
Proposed mechanisms of action for systemic and remote PBM. Abbreviations: PBM, photobiomodulation; ROS, reactive oxygen species; IFNγ, interferon gamma; IL-10, interleukin 10; TGFβ-1, transforming growth factor beta 1; IGF-1, insulin-like growth factor 1; BNDF, brain-derived neurotrophic factor; Aβ, amyloid beta; PGC1-α, peroxisome proliferator-activated receptor gamma coactivator 1-alpha; FNDC5, fibronectin type III domain-containing protein 5

Another recent study reported the potential benefits of employing abdomen-targeted PBM therapy for AD [32] (Fig. 4). In this study, wild-type mice were infused intracerebroventricularly with Aβ and then treated sequentially with 630 nm, 730 nm, and 850 nm abdomen-targeted PBM (continuous wave, 100 J/cm^2^ daily, 5 times a week for 8 weeks). The results demonstrated that PBM at all three wavelengths significantly alleviated Aβ infusion-induced memory impairment, along with reduced amyloidosis and tau phosphorylation in the hippocampus [32]. Moreover, abdomen-targeted PBM led to remarkable alterations in the expression levels of more than 500 proteins involved in hormone synthesis, phagocytosis, and metabolism in the hippocampus [32]. The findings of a 16S rRNA gene sequencing study further revealed changes in the diversity and abundance of the intestinal flora in AD mice following abdominal irradiation with PBM. The PBM-treated microbiome aligned more closely with the composition of the intestinal microflora in healthy animals [32].

Given that light could penetrate the abdominal region and affect intestinal tissues, abdominal irradiation could be a promising route for PBM delivery, and may potentially benefit AD through the gut-brain axis. Nevertheless, further studies are needed to determine any potential adverse effects and establish a safe dosage.

### Lymph node-targeted PBM

Recently, immunotherapy for AD has garnered increasing research interest [134]. CD4 + T cells (commonly known as helper T cells) are one of the key targets in these investigations because they play a pivotal role in the immune response. Once activated in the lymph nodes, CD4 cells can be further recruited into other organs including the brain, where they influence various cellular processes by releasing regulatory cytokines and affecting glial cell activity [135, 136]. Studies have shown that early temporary depletion of regulatory T cells worsens cognitive impairment in experimental mouse models of AD. Conversely, increasing regulatory T cells through peripheral regulation demonstrated the potential to mitigate AD-associated cognitive deficits [137]. This highlights the potential of targeting peripheral immune cell regulation to treat CNS diseases.

A recent study explored the therapeutic potential of PBM targeted to axillary lymph nodes for AD [33] (Fig. 4). When PBM (635 nm, continuous wave, 2 J/cm^2^ at lymph node daily) was applied to the axillary lymph nodes for one month, increased adult hippocampal neurogenesis and fewer cognitive deficits were observed in both APP/PS1 and 3xTg-AD mice. Mechanistically, lymph node-targeted PBM induced reactive oxygen species (ROS) in T cells, thus activating the JAK2/STAT4/STAT5 signaling pathway. This led to increased levels of IFN-γ/IL-10 in non-parenchymal CD4 + T cells [33]. The infiltration of these non-parenchymal CD4 + T cells into the brain subsequently increased the expression of immune mediators IFN-γ/IL-10 in brain tissue, alleviating neuroinflammation and reactive astrogliosis. Additionally, PBM increased the expression levels of TGFβ1/IGF-1/BDNF in the brain [33]. These changes may create an improved microenvironment and foster adult hippocampal neurogenesis. Moreover, culturing neural stem cells derived from AD mice with PBM-treated T lymphocyte-conditioned medium enhanced cell differentiation [33]. These findings suggest a new route for the application of PBM, which could be useful for therapeutic purposes.

### Skeletal muscle-targeted PBM

Irisin is a recently discovered myokine, which has garnered considerable attention for its neuroprotective properties [138–140]. Myokines are polypeptides that are produced, expressed, and released by muscle fibers, and exert autocrine, paracrine, or endocrine effects elsewhere in the body. Physical exercise induces an increase in the transcriptional coactivator PGC-1α in skeletal muscle, leading to elevated expression of fibronectin type III domain-containing protein 5 (FNDC5), which is the membrane-bound precursor of irisin [138]. This precursor is subsequently cleaved into irisin. Circulating irisin can penetrate the blood–brain barrier, where it exerts neuroprotective effects. Irisin is believed to play a pivotal role in the protective mechanisms of physical exercise against AD [141, 142]. Recombinant irisin, administered either intracerebroventricularly or peripherally, was demonstrated to mitigate synaptic deficits and memory impairment in both transgenic AD mice and mice infused with Aβ [141, 142]. Similar results have been reported with the intracerebroventricular injection of an FNDC5 encoding adenoviral vector [141].

Importantly, PBM can also activate PGC-1α or increase irisin production when used to irradiate skeletal muscle [143, 144]. Following PBM treatment (0.6 J and 5 J), upregulation of PGC-1α expression was observed in dystrophic primary muscle cells, with no associated cytotoxicity [144]. Gastrocnemius muscle-targeted PBM (808 nm, continuous wave) applied daily for 2 months resulted in a significant increase in muscle irisin levels, comparable to the effects of 2 months of swimming training [143]. While the effects of skeletal muscle-targeted PBM are under investigation, these preliminary studies suggest the hypothesis that skeletal muscle-targeted PBM may benefit AD by activating PGC1-α/FNDC5 and subsequently producing myokines like irisin. The proposed mechanism is illustrated in Fig. 4.

## Systemic PBM therapy for AD

Currently, only a few studies have explored the therapeutic efficacy of systemic PBM for AD. Using a whole-body PBM device fitted with an LED array, one research group investigated the effect of a 60-day PBM treatment (1070 ± 50 nm, pulsed wave, 10 and 40 Hz, 4.5 J/cm^2^ daily) for AD (Fig. 4) [25]. The results showed that when applied to the entire body of APP/PS1 mice, there was a notable reduction in Aβ burden in the hippocampus and the cortex [25]. Subsequent experiments revealed that PBM modulated the activation of microglial cells to increase their capacity for the engulfment of Aβ plaques. Additionally, there was a decrease in perivascular pro-inflammatory microglia and an improvement in vessel density post-PBM treatment [25]. Further analysis using Pearson correlation coefficients showed a strongly negative correlation between Aβ burden and vessel density and length. The increased vessel length was also associated with better performance in memory-associated behavioral tests. Notably, a more significant therapeutic benefit was observed with 10 Hz pulsed light compared to continuous wave light, although the underlying mechanism remains unknown.

In summary, this preliminary investigation provided initial evidence supporting the potential of systemic PBM therapy for AD. However, considering that exposing multiple tissues to light may induce a variety of biological effects, further studies are warranted to elucidate the potential mechanism of any crosstalk.

## Challenges and solutions

### Light penetration and its measurement

In both laboratory studies and clinical practice, transcranial PBM remains the most widely used paradigm. Despite the direct application of light to the head, only a small fraction of the irradiated light reaches the brain tissue due to losses caused by its passage through multiple layers, including the dura, meninges, periosteum, scalp, and skull bone [12]. The inherent differences in the physiological structures of human and laboratory animal skulls make it challenging to extrapolate results from experimental animals to clinical measurements in humans. This fact limits the clinical application of PBM, as there is currently no consensus on the exact transmittance of PBM light through the human brain or the recommended PBM dose received at different brain regions.

The measurement of transcranial PBM in experimental animals typically involves isolating the animal head, cross-sectioning it to expose specific layers such as the cortex and hippocampus, and administering PBM onto the isolated head through the skin and skull. The power value at different depths is then quantified using an optical power meter [51, 145]. However, comparing the skulls of humans to animals reveals a significant attenuation of light energy penetration [146]. The measurement of light emission is also a critical aspect to consider, as it could significantly influence light penetration and absorption [147]. However, the absence of standardized measures of light emission across studies introduces significant variability, stemming from differences in devices and methodologies employed. This variability makes it difficult to directly compare and evaluate the findings across different studies. Consequently, further research is necessary to comprehensively evaluate the biological effects and therapeutic efficacy of PBM using standardized equipment.

A pilot study utilized a chick embryo model situated beneath a human scalp and skull to explore the biological impact of various laser and LED stimulation systems. The findings revealed that a 10-min session of yellow laser stimulation (at 589 nm) directed through a human skull led to a notable increase in blood volume in the chick embryo model [148]. This finding implies the potential for light penetration through the human skull to induce biological responses [148]. Using an intact human skull, another study investigated the transmission capabilities of various lasers [149]. Remarkably, alongside red (658 nm) and infrared (810 nm) lasers, yellow lasers (589 nm, 50 mW) also exhibited the ability to penetrate the human skull [149]. Moreover, in a formalin-preserved cadaveric model, using an 830 nm LED light source was reported to penetrate soft tissue, bone, and brain parenchyma, but the transmission was only 0.9% [150]. Another pivotal study investigated transcranial light penetration in intact human cadaver heads. The findings suggested that 808 nm wavelength light applied transcranially could penetrate the scalp, skull, and meninges, reaching a depth in the brain of approximately 40–50 mm [151]. While NIR light of other wavelengths can also penetrate the human skull to reach brain tissues, their penetration depths remain relatively low, posing challenges for reaching deeper brain regions [152, 153]. Notably, the penetration of NIR light appears to differ between living and postmortem tissue, emphasizing the limitations inherent in the determination of light penetration obtained using human cadaveric experiments [153].

While the extent to which transcranial PBM can penetrate the human skull may be restricted, several human studies have verified its direct impact on brain activity. For instance, research has shown that transcranial PBM can elicit responses in various brain regions, such as the putamen, primary somatosensory cortex, and parietal association cortex [154]. Moreover, transcranial PBM has been demonstrated to enhance the power of alpha, beta, and gamma brain waves as measured by electroencephalography [155]. The beneficial effects of transcranial PBM using different wavelengths have also been widely reported in patients with various neurological disorders including stroke, traumatic brain injury, AD, and other types of dementia [156–158]. Nevertheless, the failure to measure the light penetration through the skull to specific brain tissue regions hampers accurate dose evaluation in PBM and may hinder its future clinical application. A feasible solution may involve evaluating the penetration of light and measuring the power output with different PBM parameters in fresh, unfixed human cadaver tissue. This approach could establish a reliable foundation for determining the appropriate dosage for PBM therapy. Another option is to develop human head-mimicking models that closely resemble the anatomical and physiological characteristics of the human head, and integrate implantable optical power sensors for more precise evaluation. Similar challenges need to be overcome in remote PBM therapy. However, since this treatment paradigm is still in its infancy, research on remote PBM should prioritize evaluating its efficacy and understanding its biological mechanisms in experimental animal models.

Intriguingly, recent studies have suggested a novel approach to stimulate deeper brain regions by combining NIR laser with photosensitive nanoparticles [159]. When exposed to optical stimulation from an NIR laser, designed nanodrugs can be released from the photosensitive nanomaterials to targeted brain regions, allowing for more precise regulation of neural activity and stimulation of deeper brain regions [159]. Several studies have demonstrated the efficacy of this therapy combination for AD [160, 161]. This topic has been elegantly reviewed elsewhere [159, 162, 163] and is beyond the scope of this review. Nevertheless, despite the promising initial findings, the safety of this combination therapy requires further investigation.

### Biphasic dose–response and determination of the clinical dose

The biphasic dose–response phenomenon describes a non-linear relationship between the applied dose and the resulting biological effects. This is where any treatment induces a beneficial response at a low dose but elicits an inhibitory response at a much higher dose. Notably, these effects (also known as hormesis) have been observed in PBM therapy [164, 165]. In cultured cortical neurons, exposure to 810 nm PBM at 25 mW/cm^2^ resulted in different outcomes at different fluences [166]. At a fluence of 3 J/cm^2^, a maximal increase in ATP production and mitochondrial membrane potential was observed. Conversely, a fluence of 30 J/cm^2^ produced inhibitory effects, causing damage to mitochondria, while lower fluences (0.03 and 0.3 J/cm^2^) did not induce any discernible biological effects [166]. An experimental study in rats also demonstrated the biphasic dose–response effect of transcranial PBM on CCO activity [167]. CCO activity increased by 14% at a lower dose of 10.9 J/cm^2^, 10% at the higher dose of 21.6 J/cm^2^, and only 3% at the highest dose of 32.9 J/cm^2^ [167]. Consequently, further research is required to assess the effective dose of transcranial PBM before widespread clinical application.

Based on current knowledge obtained from experimental animal studies, postmortem studies, and Monte Carlo simulation, the proposed effective clinical dose for transcranial PBM at the cortical level is 5–10 J/cm^2^ [15]. Notably, several non-human primate models have been established in recent years [168, 169]. Given the resemblance in neuroanatomical structure and higher-order cognitive functions between non-human primates and humans, it is conceivable that the use of these experimental animal models will contribute significantly to evaluating the effective dose and therapeutic value of transcranial PBM for AD.

### Light delivery approach

While the effectiveness of various PBM treatment paradigms for AD-associated pathologies has been explored, little is known about potential differences when light is irradiated onto different tissues. In this sense, further research is required to compare and evaluate the therapeutic efficacy of different PBM paradigms, which may offer valuable insights into the optimal approach for light delivery or alternative treatment options.

Moreover, considering the varied biological effects induced by PBM when applied to different tissues, the combination of multiple different light delivery approaches may potentially yield better results compared to a single light delivery paradigm. A modified PBM technique, involving irradiation onto both the head (850 nm, and 650 nm, pulsed wave, 10 Hz, 8.4 J/cm^2^ at the skin level daily for 7 days) and the abdomen (850 nm and 650 nm, pulsed wave, 10 Hz, 8.4 J/cm^2^ at the skin level daily for 7 days), resulted in reductions in Aβ, tau, markers of oxidative stress, apoptosis, and inflammation in mice intracerebroventricularly infused with Aβ, as reported by a research group from France [31]. In another case report, the use of three different wearable LED devices, including a transcranial light helmet (635 nm, continuous wave), a body pad (810 nm, continuous wave), and an intranasal LED device (810 nm, pulsed wave, 10 Hz), used at the same time improved cognitive function in a patient with a history of AD [170]. A case series report demonstrated that the combination of transcranial and intranasal PBM (810 nm, pulsed wave, 10 Hz, 375 or 639 J per week) for 12 weeks significantly improved cognition in five patients with mild to moderately severe cognitive impairment [20]. However, the improved cognitive function conferred by PBM was significantly diminished during the follow-up period (week 16), indicating that continuous application may be necessary to maintain clinical improvements [20]. However, it’s crucial to consider the limitations of these case reports, including small sample sizes and lack of control groups, which may limit the generalizability and robustness of the observed outcomes. Further well-designed trials with larger cohorts and rigorous controls are required. In this aspect, a randomized, double-blind, and sham-controlled trial provided robust evidence for the therapeutic value of PBM in cognitive improvement [18]. In this study, 53 mild-to-moderate AD patients were randomly assigned to receive either head-abdomen PBM treatment (40 treatment sessions lasting 25 min each over 8 weeks) or were allocated to a sham treatment group [18]. Following the intervention, patients who received PBM therapy reported improved cognitive performances, measured by neuropsychological tests during the 4-week follow-up assessment, with minimal adverse effects recorded [18]. Furthermore, while safety aspects require further evaluation, another report highlighted the therapeutic potential of intravascular PBM for AD [171]. Under local anesthesia, the common femoral artery of patients was catheterized, and a thin, flexible fiber-optic cable was advanced to the distal sections of the anterior and middle cerebral arteries, where light was irradiated (632 nm, 25 mW, 20–40 min per session). After 6–12 months of daily treatment, improved cerebral microcirculation and metabolism, as well as less cognitive impairment, were reported [171]. These promising findings suggest that the combined application of different PBM paradigms may result in synergistic effects, which remain to be determined in future research. Further investigation into the therapeutic potential of alternative light delivery approaches is therefore warranted.

### Clinical translation and application of PBM

In the past decade, considerable advances have been achieved in the use of PBM therapy for AD. Still, several challenges remain to be resolved before the widespread clinical application of this therapy. Research conducted in experimental animal models has significantly advanced our comprehension of the biological mechanisms underlying the beneficial effects of PBM for AD, particularly transcranial PBM therapy. While the beneficial effects of PBM are widely recognized, the precise molecular mechanisms driving these effects are still not fully understood. Historically, CCO has been implicated as the primary target for this therapy, as discussed in the previous sections. However, this foundational hypothesis lacks direct empirical support. Moreover, given the reported various biological effects conferred by PBM, how photoactivated CCO may potentially interact with these cellular processes remains to be determined. A better understanding of the fundamental mechanism of PBM will provide insight into the rational design and application of this therapy.

Despite the widely reported beneficial effects of PBM on animal models of AD, a recent study [172] found no significant impact of PBM. In this randomized, blinded study, 5xFAD mice received transcranial PBM treatment (810 nm, pulsed wave at 100 Hz, three times a week) from 1 month old to 6 months old. The results revealed no discernible differences in memory performance, amyloid load, neuronal loss, or microglial response between the 5xFAD mice treated with PBM and the control group [172]. This outcome may be attributed to variations in light parameters/treatment dosage and the devices utilized. It is worth noting that many animal studies neglect to report crucial light parameters such as light power output on target tissues and waveform, potentially leading to discrepancies even when employing the same light wavelength. Therefore, the findings of these preclinical studies should be approached with caution, and more standardized studies are required in the future.

Further evaluation of the penetration of different light parameters through the skull to reach brain tissue and the effects of power output on specific brain regions is warranted. Potential approaches include measuring these parameters in fresh, unfixed human cadavers or human head-mimicking models, as discussed earlier, which could provide better control over the effective light dose. Despite the proposal of a variety of light delivery approaches, their efficacy, mechanisms of action, and safety must be thoroughly investigated in experimental animals before any clinical application, making this a priority. The therapeutic potential of the combination of various light delivery approaches also warrants further validation in animal models.

The therapeutic potential of transcranial PBM for AD has been documented in various clinical trials and case reports, where it has demonstrated only minimal side effects. However, given the observed biphasic dose response in cultured neural cells and animal models, coupled with the complexity of AD pathogenesis, it is crucial to conduct further investigation to determine the effective dose of transcranial PBM in large animal models that are both physiologically and anatomically nearer to humans. In this context, recently developed non-human primate models of AD may serve as a tool for addressing these concerns and may provide valuable insight into clinical applications.

Furthermore, it is crucial to acknowledge that the majority of preclinical studies have primarily involved rodent models. The structural and anatomical differences between these experimental animals and human beings pose a challenge in translating these findings from the laboratory into clinical practice. The discrepancy in brain size between small experimental animals also presents a significant hurdle for translating this therapy. While light can penetrate the entire brain of animals, reaching deeper brain regions like the dorsal hippocampus and amygdala [145], achieving similar coverage in humans is challenging, with most light only reaching the cortical level. Consequently, the doses administered and resulting biological effects may vary significantly between rodents and humans. The full extent of PBM’s effects on a human scale remains to be determined. In this regard, some completed randomized controlled trials may offer valuable insights into the therapeutic efficacy of this therapy in humans. In a small randomized controlled trial, thirty-two dementia patients were randomly assigned to either receive 8 weeks of transcranial PBM therapy or a sham intervention. The evaluation revealed that those who received transcranial PBM showed improved cognitive symptoms compared to those who underwent the sham procedure [173]. Similarly, another randomized clinical trial investigated the impact of combined PBM and aerobic exercise on cognitive function in elderly AD patients [174]. Sixty elderly AD patients were randomly divided into two groups: one receiving PBM (intravascular and intranasal PBM, 30 min per session, twice a day, three days a week) alongside moderate-intensity aerobic exercise, and the other receiving a placebo intervention in addition to aerobic exercise over 12 weeks. Results indicated that patients who received PBM alongside moderate-intensity aerobic exercise exhibited significant improvements in cognition, life quality, and balance function compared to the control group receiving placebo therapy alongside aerobic exercise [174]. Moreover, the most robust evidence stems from a randomized and double-blind trial, demonstrating that a two-month head-abdomen PBM treatment yielded slight cognitive improvement, as discussed in previous sections [18]. Although the treatment duration was relatively short compared to current clinical trials on AD therapeutic intervention, typically lasting 18 months [175, 176], this preliminary trial is encouraging as it suggests that a relatively short treatment period for this therapy may be sufficient to produce favorable outcomes to some extent. Furthermore, no obvious adverse events were reported in these studies, suggesting this therapy may have a better safety and tolerability profile compared to conventional pharmacological methods. Nevertheless, to confirm the efficacy and determine the optimal dosage of this therapy in AD, it is crucial to conduct further large-scale, methodologically rigorous, randomized controlled trials involving a more extensive patient population. Moreover, extending the treatment duration is essential for a comprehensive evaluation of its overall effects. Several registered clinical trials are currently underway (jRCTs032230339, NCT05926011, NCT04784416, NCT03484143), involving multi-site participation, larger sample sizes, and longer treatment durations, which are expected to provide additional insights into the clinical application of PBM.

Notably, it may be unable to make an effective comparison of therapeutic efficacy in different studies due to the unstandardized instruments and measurement methods. Indeed, based on different parameters such as wavelengths, wave mode (continuous wave and pulsed wave), irradiance, fluence, and treatment protocols, the effects of PBM treatments can vary, which may yield different outcomes. Thus, PBM may not be considered a standardized treatment with guaranteed safety and efficacy across the board. Each specific combination of parameters and protocols needs to be evaluated individually as a unique medical device.

**Fig. 5:**
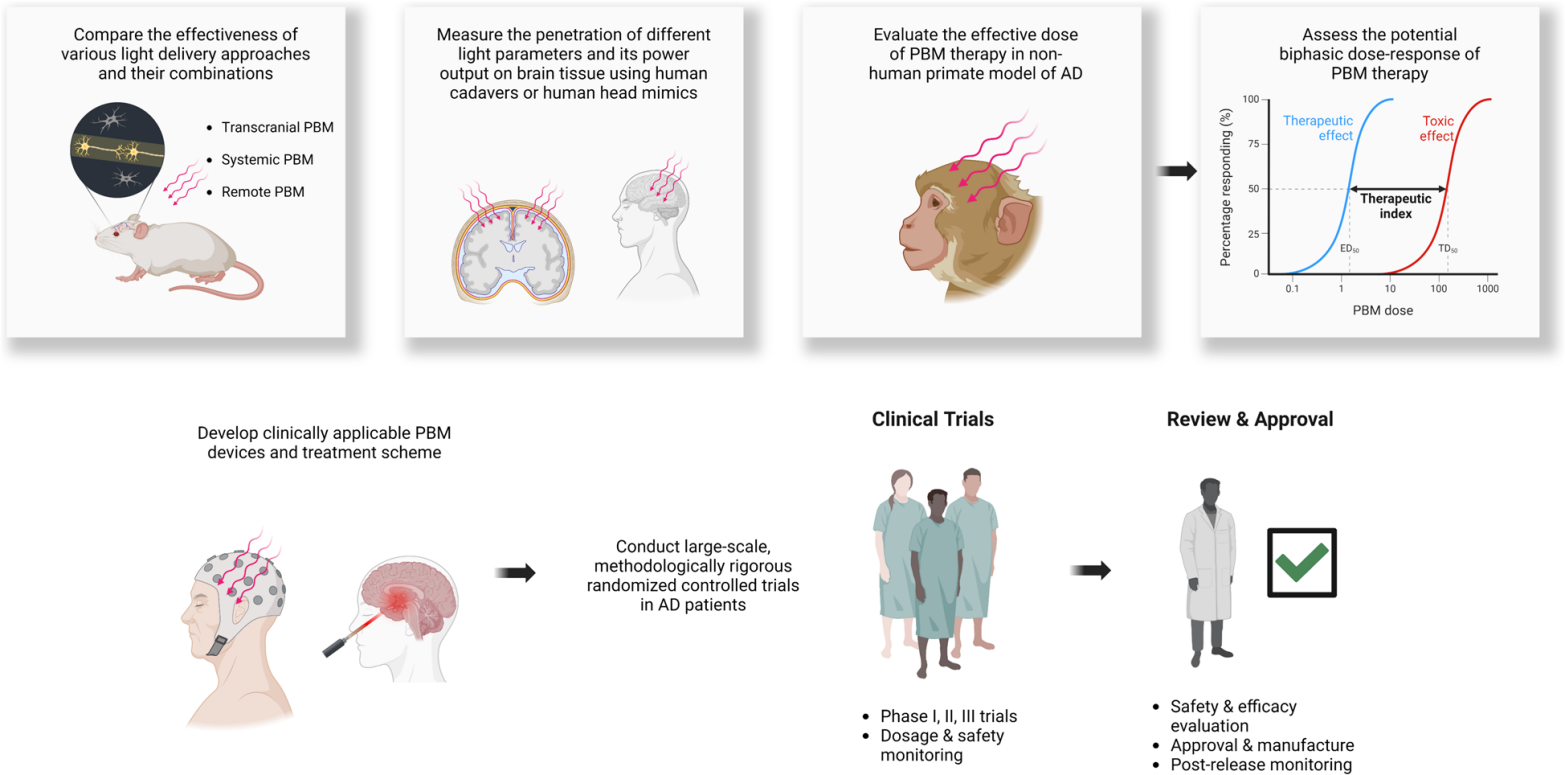
Summary of directions for future research and the clinical translation of PBM therapy. Abbreviations: PBM, photobiomodulation; AD, Alzheimer’s disease; CSF, cerebrospinal fluid

Given the lengthy pre-clinical asymptomatic phase of AD and the limited effectiveness of existing therapies for AD patients, preventive intervention has emerged as a promising approach for AD management [177, 178]. Technological advancements have led to the identification of several early biomarkers of AD, offering the potential for early disease detection and extending the window for timely intervention [179–181]. Advances in neuroimaging techniques will also enable precise neurological and structural evaluation, potentially allowing for the identification of early pathological alterations associated with AD [182]. To date, clinical trials in this field have predominantly focused on patients with confirmed AD. Yet, with advancements in identifying early AD pathological changes and refining safe treatment protocols from subsequent trials, it would be attractive to explore if early PBM treatment could slow down the progression of AD, which may be more clinically meaningful and may offer better control over the overall disease burden (Fig. 5). Future studies into this field could potentially pave the way for a novel direction in the management of AD.

## Conclusions

The multifactorial nature of AD and its complex pathogenesis underscore the need for multimodal and individualized treatment interventions. As a non-invasive and non-pharmacological intervention, PBM therapy, especially transcranial PBM, has shown the potential to prevent and/or alleviate AD-associated pathology and cognitive decline. Current knowledge suggests that PBM can activate various signaling pathways within the brain, exerting a variety of beneficial biological effects. Accumulated evidence from both experimental animal studies and clinical trials supports its potential in AD management, with only minimal side effects. Thus, it is time to begin to translate this promising therapy from the laboratory bench to the bedside. Importantly, while existing evidence suggests the potential of PBM as a therapeutic intervention for AD, further clinical trials are imperative to thoroughly assess its efficacy and establish treatment protocols. Such endeavors hold promise of introducing novel opportunities for the safe and effective management of AD.

## Data Availability

Not applicable.

## References

[1] Scheltens P, De Strooper B, Kivipelto M, Holstege H, Chetelat G, Teunissen CE (2021). Alzheimer’s disease. Lancet.

[2] Gillis C, Montenigro P, Nejati M, Maserejian N (2023). Estimating prevalence of early Alzheimer’s disease in the United States, accounting for racial and ethnic diversity. Alzheimers Dement.

[3] Lane CA, Hardy J, Schott JM (2018). Alzheimer’s disease. Eur J Neurol.

[4] 2023 Alzheimer’s disease facts and figures. Alzheimers Dement. 2023;19:1598–695. 10.1002/alz.13016 36918389

[5] Zhang Y, Chen H, Li R, Sterling K, Song W (2023). Amyloid beta-based therapy for Alzheimer’s disease: challenges, successes and future. Signal Transduct Target Ther.

[6] Huang Z, Jordan JD, Zhang Q (2023). Myelin pathology in Alzheimer’s disease: potential therapeutic opportunities. Aging Dis.

[7] Cummings J, Osse AML, Cammann D, Powell J, Chen J (2024). Anti-amyloid monoclonal antibodies for the treatment of Alzheimer’s disease. BioDrugs.

[8] Cummings J, Zhou Y, Lee G, Zhong K, Fonseca J, Cheng F (2023). Alzheimer’s disease drug development pipeline: 2023. Alzheimers Dement (N Y).

[9] Ibrahim MM, Gabr MT (2019). Multitarget therapeutic strategies for Alzheimer’s disease. Neural Regen Res.

[10] Albertini C, Salerno A, de SenaMurteira Pinheiro P, Bolognesi ML (2021). From combinations to multitarget-directed ligands: A continuum in Alzheimer’s disease polypharmacology. Med Res Rev.

[11] Chung H, Dai T, Sharma SK, Huang YY, Carroll JD, Hamblin MR (2012). The nuts and bolts of low-level laser (light) therapy. Ann Biomed Eng.

[12] Salehpour F, Mahmoudi J, Kamari F, Sadigh-Eteghad S, Rasta SH, Hamblin MR (2018). Brain photobiomodulation therapy: a narrative review. Mol Neurobiol.

[13] Hamblin MR, Salehpour F (2021). Photobiomodulation of the brain: shining light on Alzheimer’s and other neuropathological diseases. J Alzheimers Dis.

[14] Cardoso FDS, Gonzalez-Lima F, Gomes da Silva S (2021). Photobiomodulation for the aging brain. Ageing Res Rev.

[15] Enengl J, Hamblin MR, Dungel P (2020). Photobiomodulation for Alzheimer’s disease: translating basic research to clinical application. J Alzheimers Dis.

[16] Hamblin MR (2016). Shining light on the head: Photobiomodulation for brain disorders. BBA Clin.

[17] Yang L, Wu C, Parker E, Li Y, Dong Y, Tucker L (2022). Non-invasive photobiomodulation treatment in an Alzheimer disease-like transgenic rat model. Theranostics.

[18] Blivet G, Relano-Gines A, Wachtel M, Touchon J (2022). A randomized, double-blind, and sham-controlled trial of an innovative brain-gut photobiomodulation therapy: safety and patient compliance. J Alzheimers Dis.

[19] Baik JS, Lee TY, Kim NG, Pak K, Ko SH, Min JH (2021). Effects of photobiomodulation on changes in cognitive function and regional cerebral blood flow in patients with mild cognitive impairment: a pilot uncontrolled trial. J Alzheimers Dis.

[20] Saltmarche AE, Naeser MA, Ho KF, Hamblin MR, Lim L (2017). Significant improvement in cognition in mild to moderately severe dementia cases treated with transcranial plus intranasal photobiomodulation: case series report. Photomed Laser Surg.

[21] Gordon LC, Johnstone DM (2019). Remote photobiomodulation: an emerging strategy for neuroprotection. Neural Regen Res.

[22] Hamblin MR (2019). Photobiomodulation for Alzheimer’s disease: has the light dawned?. Photonics.

[23] Monteiro F, Carvalho O, Sousa N, Silva FS, Sotiropoulos I (2022). Photobiomodulation and visual stimulation against cognitive decline and Alzheimer’s disease pathology: a systematic review. Alzheimers Dement (N Y).

[24] Lee TL, Ding Z, Chan AS (2023). Can transcranial photobiomodulation improve cognitive function? A systematic review of human studies. Ageing Res Rev.

[25] Tao L, Liu Q, Zhang F, Fu Y, Zhu X, Weng X (2021). Microglia modulation with 1070-nm light attenuates Abeta burden and cognitive impairment in Alzheimer’s disease mouse model. Light Sci Appl.

[26] Wang M, Cao J, Amakye WK, Gong C, Li Q, Ren J (2020). Mid infrared light treatment attenuates cognitive decline and alters the gut microbiota community in APP/PS1 mouse model. Biochem Biophys Res Commun.

[27] Bowen R, Arany PR (2023). Use of either transcranial or whole-body photobiomodulation treatments improves COVID-19 brain fog. J Biophotonics.

[28] Fitzmaurice B, Heneghan NR, Rayen A, Soundy A (2022). Whole-body photobiomodulation therapy for chronic pain: a protocol for a feasibility trial. BMJ Open.

[29] Gordon LC, Martin KL, Torres N, Benabid AL, Mitrofanis J, Stone J (2023). Remote photobiomodulation targeted at the abdomen or legs provides effective neuroprotection against parkinsonian MPTP insult. Eur J Neurosci.

[30] Kim B, Brandli A, Mitrofanis J, Stone J, Purushothuman S, Johnstone DM (2017). Remote tissue conditioning - an emerging approach for inducing body-wide protection against diseases of ageing. Ageing Res Rev.

[31] Blivet G, Meunier J, Roman FJ, Touchon J (2018). Neuroprotective effect of a new photobiomodulation technique against Abeta(25–35) peptide-induced toxicity in mice: Novel hypothesis for therapeutic approach of Alzheimer’s disease suggested. Alzheimers Dement (N Y).

[32] Chen Q, Wu J, Dong X, Yin H, Shi X, Su S (2021). Gut flora-targeted photobiomodulation therapy improves senile dementia in an Ass-induced Alzheimer’s disease animal model. J Photochem Photobiol B.

[33] Wu X, Shen Q, Chang H, Li J, Xing D (2022). Promoted CD4(+) T cell-derived IFN-gamma/IL-10 by photobiomodulation therapy modulates neurogenesis to ameliorate cognitive deficits in APP/PS1 and 3xTg-AD mice. J Neuroinflammation.

[34] Karu TI, Pyatibrat LV, Kalendo GS (2004). Photobiological modulation of cell attachment via cytochrome c oxidase. Photochem Photobiol Sci.

[35] AhamedBasha A, Mathangi DC, Shyamala R (2016). Effect of LED photobiomodulation on fluorescent light induced changes in cellular ATPases and Cytochrome c oxidase activity in Wistar rat. Lasers Med Sci.

[36] Wikstrom M, Krab K, Sharma V (2018). Oxygen activation and energy conservation by cytochrome c oxidase. Chem Rev.

[37] Cooper CE, Cope M, Springett R, Amess PN, Penrice J, Tyszczuk L (1999). Use of mitochondrial inhibitors to demonstrate that cytochrome oxidase near-infrared spectroscopy can measure mitochondrial dysfunction noninvasively in the brain. J Cereb Blood Flow Metab.

[38] de Freitas LF, Hamblin MR (2016). Proposed mechanisms of photobiomodulation or low-level light therapy. IEEE J Sel Top Quantum Electron.

[39] Hamblin MR (2018). Mechanisms and mitochondrial redox signaling in photobiomodulation. Photochem Photobiol.

[40] Lima PLV, Pereira CV, Nissanka N, Arguello T, Gavini G, Maranduba C (2019). Photobiomodulation enhancement of cell proliferation at 660 nm does not require cytochrome c oxidase. J Photochem Photobiol B.

[41] Guo T, Zhang D, Zeng Y, Huang TY, Xu H, Zhao Y (2020). Molecular and cellular mechanisms underlying the pathogenesis of Alzheimer’s disease. Mol Neurodegener.

[42] Tiwari S, Atluri V, Kaushik A, Yndart A, Nair M (2019). Alzheimer’s disease: pathogenesis, diagnostics, and therapeutics. Int J Nanomedicine.

[43] Purushothuman S, Johnstone DM, Nandasena C, Mitrofanis J, Stone J (2014). Photobiomodulation with near infrared light mitigates Alzheimer’s disease-related pathology in cerebral cortex - evidence from two transgenic mouse models. Alzheimers Res Ther.

[44] Zhang L, Xing D, Zhu D, Chen Q (2008). Low-power laser irradiation inhibiting Abeta25-35-induced PC12 cell apoptosis via PKC activation. Cell Physiol Biochem.

[45] Liang J, Liu L, Xing D (2012). Photobiomodulation by low-power laser irradiation attenuates Abeta-induced cell apoptosis through the Akt/GSK3beta/beta-catenin pathway. Free Radic Biol Med.

[46] Chasseigneaux S, Allinquant B (2012). Functions of Abeta, sAPPalpha and sAPPbeta : similarities and differences. J Neurochem.

[47] O’Brien RJ, Wong PC (2011). Amyloid precursor protein processing and Alzheimer’s disease. Annu Rev Neurosci.

[48] Bandyopadhyay S, Goldstein LE, Lahiri DK, Rogers JT (2007). Role of the APP non-amyloidogenic signaling pathway and targeting alpha-secretase as an alternative drug target for treatment of Alzheimer’s disease. Curr Med Chem.

[49] Kojro E, Fahrenholz F (2005). The non-amyloidogenic pathway: structure and function of alpha-secretases. Subcell Biochem.

[50] Dobrowolska JA, Michener MS, Wu G, Patterson BW, Chott R, Ovod V (2014). CNS amyloid-beta, soluble APP-alpha and -beta kinetics during BACE inhibition. J Neurosci.

[51] Zhang Z, Shen Q, Wu X, Zhang D, Xing D (2020). Activation of PKA/SIRT1 signaling pathway by photobiomodulation therapy reduces Abeta levels in Alzheimer’s disease models. Aging Cell.

[52] Rizzi L, Roriz-Cruz M (2018). Sirtuin 1 and Alzheimer’s disease: An up-to-date review. Neuropeptides.

[53] Mehramiz M, Porter T, O’Brien EK, Rainey-Smith SR, Laws SM (2023). A potential role for sirtuin-1 in Alzheimer’s disease: reviewing the biological and environmental evidence. J Alzheimers Dis Rep.

[54] Mehramiz M, Porter T, Laws SM, Rainey-Smith SR (2022). Sleep, sirtuin 1 and Alzheimer’s disease: a review. Aging Brain.

[55] Julien C, Tremblay C, Emond V, Lebbadi M, Salem N, Bennett DA (2009). Sirtuin 1 reduction parallels the accumulation of tau in Alzheimer disease. J Neuropathol Exp Neurol.

[56] Zhang M, Tang Z (2023). Therapeutic potential of natural molecules against Alzheimer’s disease via SIRT1 modulation. Biomed Pharmacother.

[57] Sancho-Balsells A, Borras-Pernas S, Flotta F, Chen W, Del Toro D, Rodriguez MJ (2024). Brain-gut photobiomodulation restores cognitive alterations in chronically stressed mice through the regulation of Sirt1 and neuroinflammation. J Affect Disord.

[58] Cho GM, Lee SY, Park JH, Kim MJ, Park KJ, Choi BT (2020). Photobiomodulation using a low-level light-emitting diode improves cognitive dysfunction in the 5XFAD mouse model of Alzheimer’s disease. J Gerontol A Biol Sci Med Sci.

[59] Shen Q, Liu L, Gu X, Xing D (2021). Photobiomodulation suppresses JNK3 by activation of ERK/MKP7 to attenuate AMPA receptor endocytosis in Alzheimer’s disease. Aging Cell.

[60] Selkoe DJ (2002). Alzheimer’s disease is a synaptic failure. Science.

[61] Shankar GM, Walsh DM (2009). Alzheimer’s disease: synaptic dysfunction and Abeta. Mol Neurodegener.

[62] Bencsik N, Oueslati Morales CO, Hausser A, Schlett K (2023). Endocytosis of AMPA receptors: role in neurological conditions. Prog Mol Biol Transl Sci.

[63] Beattie EC, Carroll RC, Yu X, Morishita W, Yasuda H, von Zastrow M (2000). Regulation of AMPA receptor endocytosis by a signaling mechanism shared with LTD. Nat Neurosci.

[64] Bredt DS, Nicoll RA (2003). AMPA receptor trafficking at excitatory synapses. Neuron.

[65] Diering GH, Huganir RL (2018). The AMPA receptor code of synaptic plasticity. Neuron.

[66] Zhao WQ, Santini F, Breese R, Ross D, Zhang XD, Stone DJ (2010). Inhibition of calcineurin-mediated endocytosis and alpha-amino-3-hydroxy-5-methyl-4-isoxazolepropionic acid (AMPA) receptors prevents amyloid beta oligomer-induced synaptic disruption. J Biol Chem.

[67] Zhang Y, Guo O, Huo Y, Wang G, Man HY (2018). Amyloid-beta induces AMPA receptor ubiquitination and degradation in primary neurons and human brains of Alzheimer’s disease. J Alzheimers Dis.

[68] Dong Z, Han H, Li H, Bai Y, Wang W, Tu M (2015). Long-term potentiation decay and memory loss are mediated by AMPAR endocytosis. J Clin Invest.

[69] Yang L, Tucker D, Dong Y, Wu C, Lu Y, Li Y (2018). Photobiomodulation therapy promotes neurogenesis by improving post-stroke local microenvironment and stimulating neuroprogenitor cells. Exp Neurol.

[70] Wu X, Shen Q, Zhang Z, Zhang D, Gu Y, Xing D (2021). Photoactivation of TGFbeta/SMAD signaling pathway ameliorates adult hippocampal neurogenesis in Alzheimer’s disease model. Stem Cell Res Ther.

[71] Babcock KR, Page JS, Fallon JR, Webb AE (2021). Adult Hippocampal Neurogenesis in aging and Alzheimer’s disease. Stem Cell Rep.

[72] Mu Y, Gage FH (2011). Adult hippocampal neurogenesis and its role in Alzheimer’s disease. Mol Neurodegener.

[73] Mishra R, Phan T, Kumar P, Morrissey Z, Gupta M, Hollands C (2022). Augmenting neurogenesis rescues memory impairments in Alzheimer’s disease by restoring the memory-storing neurons. J Exp Med.

[74] Tropepe V, Hitoshi S, Sirard C, Mak TW, Rossant J, van der Kooy D (2001). Direct neural fate specification from embryonic stem cells: a primitive mammalian neural stem cell stage acquired through a default mechanism. Neuron.

[75] Rafalski VA, Brunet A (2011). Energy metabolism in adult neural stem cell fate. Prog Neurobiol.

[76] Meng C, He Z, Xing D (2013). Low-level laser therapy rescues dendrite atrophy via upregulating BDNF expression: implications for Alzheimer’s disease. J Neurosci.

[77] Tapia-Arancibia L, Aliaga E, Silhol M, Arancibia S (2008). New insights into brain BDNF function in normal aging and Alzheimer disease. Brain Res Rev.

[78] Feng Y, Yang L, Ma X, Huang Z, Zong X, Citadin CT (2023). Photobiomodulation treatment inhibits neurotoxic astrocytic polarization and protects neurons in in vitro and in vivo stroke models. Neurochem Int.

[79] Fear EJ, Torkelsen FH, Zamboni E, Chen KJ, Scott M, Jeffery G (2023). Use of (31) P magnetisation transfer magnetic resonance spectroscopy to measure ATP changes after 670 nm transcranial photobiomodulation in older adults. Aging Cell.

[80] Swerdlow RH (2018). Mitochondria and mitochondrial cascades in Alzheimer’s disease. J Alzheimers Dis.

[81] Sharma VK, Singh TG, Mehta V (2021). Stressed mitochondria: a target to intrude Alzheimer’s disease. Mitochondrion.

[82] Lu Y, Wang R, Dong Y, Tucker D, Zhao N, Ahmed ME (2017). Low-level laser therapy for beta amyloid toxicity in rat hippocampus. Neurobiol Aging.

[83] Ferrer I, Gomez A, Carmona M, Huesa G, Porta S, Riera-Codina M (2011). Neuronal hemoglobin is reduced in Alzheimer’s disease, argyrophilic grain disease, Parkinson’s disease, and dementia with Lewy bodies. J Alzheimers Dis.

[84] Calsolaro V, Edison P (2016). Neuroinflammation in Alzheimer’s disease: Current evidence and future directions. Alzheimers Dement.

[85] Brosseron F, Traschutz A, Widmann CN, Kummer MP, Tacik P, Santarelli F (2018). Characterization and clinical use of inflammatory cerebrospinal fluid protein markers in Alzheimer’s disease. Alzheimers Res Ther.

[86] Yang QQ, Zhou JW (2019). Neuroinflammation in the central nervous system: symphony of glial cells. Glia.

[87] Patani R, Hardingham GE, Liddelow SA (2023). Functional roles of reactive astrocytes in neuroinflammation and neurodegeneration. Nat Rev Neurol.

[88] Fakhoury M (2018). Microglia and astrocytes in Alzheimer’s disease: implications for therapy. Curr Neuropharmacol.

[89] Blasko I, Stampfer-Kountchev M, Robatscher P, Veerhuis R, Eikelenboom P, Grubeck-Loebenstein B (2004). How chronic inflammation can affect the brain and support the development of Alzheimer’s disease in old age: the role of microglia and astrocytes. Aging Cell.

[90] Guo S, Wang H, Yin Y (2022). Microglia polarization from M1 to M2 in neurodegenerative diseases. Front Aging Neurosci.

[91] Tang Y, Le W (2016). Differential roles of M1 and M2 microglia in neurodegenerative diseases. Mol Neurobiol.

[92] von Bernhardi R, Ramirez G (2001). Microglia-astrocyte interaction in Alzheimer’s disease: friends or foes for the nervous system?. Biol Res.

[93] Lian H, Litvinchuk A, Chiang AC, Aithmitti N, Jankowsky JL, Zheng H (2016). Astrocyte-microglia cross talk through complement activation modulates amyloid pathology in mouse models of Alzheimer’s disease. J Neurosci.

[94] Stepanov YV, Golovynska I, Zhang R, Golovynskyi S, Stepanova LI, Gorbach O (2022). Near-infrared light reduces beta-amyloid-stimulated microglial toxicity and enhances survival of neurons: mechanisms of light therapy for Alzheimer’s disease. Alzheimers Res Ther.

[95] Pan XD, Zhu YG, Lin N, Zhang J, Ye QY, Huang HP (2011). Microglial phagocytosis induced by fibrillar beta-amyloid is attenuated by oligomeric beta-amyloid: implications for Alzheimer’s disease. Mol Neurodegener.

[96] Koenigsknecht-Talboo J, Landreth GE (2005). Microglial phagocytosis induced by fibrillar beta-amyloid and IgGs are differentially regulated by proinflammatory cytokines. J Neurosci.

[97] Chen C, Bao Y, Xing L, Jiang C, Guo Y, Tong S (2023). Exosomes derived from M2 microglial cells modulated by 1070-nm light improve cognition in an Alzheimer’s disease mouse model. Adv Sci (Weinh).

[98] Alosco ML, Gunstad J, Jerskey BA, Xu X, Clark US, Hassenstab J (2013). The adverse effects of reduced cerebral perfusion on cognition and brain structure in older adults with cardiovascular disease. Brain Behav.

[99] Ogoh S (2017). Relationship between cognitive function and regulation of cerebral blood flow. J Physiol Sci.

[100] Alsop DC, Casement M, de Bazelaire C, Fong T, Press DZ (2008). Hippocampal hyperperfusion in Alzheimer’s disease. Neuroimage.

[101] Huang CW, Hsu SW, Chang YT, Huang SH, Huang YC, Lee CC (2018). Cerebral perfusion insufficiency and relationships with cognitive deficits in Alzheimer’s disease: a multiparametric neuroimaging study. Sci Rep.

[102] Austin BP, Nair VA, Meier TB, Xu G, Rowley HA, Carlsson CM (2011). Effects of hypoperfusion in Alzheimer’s disease. J Alzheimers Dis.

[103] Wolters FJ, Zonneveld HI, Hofman A, van der Lugt A, Koudstaal PJ, Vernooij MW (2017). Cerebral perfusion and the risk of dementia: a population-based Study. Circulation.

[104] Holmes E, Barrett DW, Saucedo CL, O’Connor P, Liu H, Gonzalez-Lima F (2019). Cognitive enhancement by transcranial photobiomodulation is associated with cerebrovascular oxygenation of the prefrontal cortex. Front Neurosci.

[105] Uozumi Y, Nawashiro H, Sato S, Kawauchi S, Shima K, Kikuchi M (2010). Targeted increase in cerebral blood flow by transcranial near-infrared laser irradiation. Lasers Surg Med.

[106] Toda N, Ayajiki K, Okamura T (2009). Cerebral blood flow regulation by nitric oxide: recent advances. Pharmacol Rev.

[107] Toda N, Okamura T (2012). Cerebral blood flow regulation by nitric oxide in Alzheimer’s disease. J Alzheimers Dis.

[108] Yokomizo S, Roessing M, Morita A, Kopp T, Ogawa E, Katagiri W (2022). Near-infrared II photobiomodulation augments nitric oxide bioavailability via phosphorylation of endothelial nitric oxide synthase. FASEB J.

[109] Yokomizo S, Kopp T, Roessing M, Morita A, Lee S, Cho S, et al. Near-infrared II photobiomodulation preconditioning ameliorates stroke injury via phosphorylation of eNOS. Stroke. 2024;Online ahead of print. 10.1161/STROKEAHA.123.045358 PMC11126363 38572660

[110] Iliff JJ, Wang M, Liao Y, Plogg BA, Peng W, Gundersen GA (2012). A paravascular pathway facilitates CSF flow through the brain parenchyma and the clearance of interstitial solutes, including amyloid beta. Sci Transl Med.

[111] Jessen NA, Munk AS, Lundgaard I, Nedergaard M (2015). The glymphatic system: a beginner’s guide. Neurochem Res.

[112] Plog BA, Nedergaard M (2018). The glymphatic system in central nervous system health and disease: past, present, and future. Annu Rev Pathol.

[113] Lohela TJ, Lilius TO, Nedergaard M (2022). The glymphatic system: implications for drugs for central nervous system diseases. Nat Rev Drug Discov.

[114] Gomolka RS, Hablitz LM, Mestre H, Giannetto M, Du T, Hauglund NL (2023). Loss of aquaporin-4 results in glymphatic system dysfunction via brain-wide interstitial fluid stagnation. Elife.

[115] Ishida K, Yamada K, Nishiyama R, Hashimoto T, Nishida I, Abe Y (2022). Glymphatic system clears extracellular tau and protects from tau aggregation and neurodegeneration. J Exp Med.

[116] Harrison IF, Ismail O, Machhada A, Colgan N, Ohene Y, Nahavandi P (2020). Impaired glymphatic function and clearance of tau in an Alzheimer’s disease model. Brain.

[117] Gu L, Dai S, Guo T, Si X, Lv D, Wang Z (2023). Noninvasive neuroimaging provides evidence for deterioration of the glymphatic system in Parkinson’s disease relative to essential tremor. Parkinsonism Relat Disord.

[118] Carotenuto A, Cacciaguerra L, Pagani E, Preziosa P, Filippi M, Rocca MA (2022). Glymphatic system impairment in multiple sclerosis: relation with brain damage and disability. Brain.

[119] Lee Y, Choi Y, Park EJ, Kwon S, Kim H, Lee JY (2020). Improvement of glymphatic-lymphatic drainage of beta-amyloid by focused ultrasound in Alzheimer’s disease model. Sci Rep.

[120] Hsu JL, Wei YC, Toh CH, Hsiao IT, Lin KJ, Yen TC (2023). Magnetic resonance images implicate that glymphatic alterations mediate cognitive dysfunction in Alzheimer disease. Ann Neurol.

[121] Li D, Liu S, Yu T, Liu Z, Sun S, Bragin D (2023). Photostimulation of brain lymphatics in male newborn and adult rodents for therapy of intraventricular hemorrhage. Nat Commun.

[122] Semyachkina-Glushkovskaya O, Klimova M, Iskra T, Bragin D, Abdurashitov A, Dubrovsky A (2021). Transcranial photobiomodulation of clearance of beta-amyloid from the mouse brain: effects on the meningeal lymphatic drainage and blood oxygen saturation of the brain. Adv Exp Med Biol.

[123] Zinchenko E, Navolokin N, Shirokov A, Khlebtsov B, Dubrovsky A, Saranceva E (2019). Pilot study of transcranial photobiomodulation of lymphatic clearance of beta-amyloid from the mouse brain: breakthrough strategies for non-pharmacologic therapy of Alzheimer’s disease. Biomed Opt Express.

[124] Yun SH, Kwok SJJ (2017). Light in diagnosis, therapy and surgery. Nat Biomed Eng.

[125] Wang M, Yan C, Li X, Yang T, Wu S, Liu Q (2024). Non-invasive modulation of meningeal lymphatics ameliorates ageing and Alzheimer’s disease-associated pathology and cognition in mice. Nat Commun.

[126] Mayer EA, Nance K, Chen S (2022). The gut-brain axis. Annu Rev Med.

[127] Jiang C, Li G, Huang P, Liu Z, Zhao B (2017). The gut microbiota and Alzheimer’s disease. J Alzheimers Dis.

[128] Guo M, Peng J, Huang X, Xiao L, Huang F, Zuo Z (2021). Gut microbiome features of chinese patients newly diagnosed with Alzheimer’s disease or mild cognitive impairment. J Alzheimers Dis.

[129] Vogt NM, Kerby RL, Dill-McFarland KA, Harding SJ, Merluzzi AP, Johnson SC (2017). Gut microbiome alterations in Alzheimer’s disease. Sci Rep.

[130] Kim MS, Kim Y, Choi H, Kim W, Park S, Lee D (2020). Transfer of a healthy microbiota reduces amyloid and tau pathology in an Alzheimer’s disease animal model. Gut.

[131] Kim N, Jeon SH, Ju IG, Gee MS, Do J, Oh MS (2021). Transplantation of gut microbiota derived from Alzheimer’s disease mouse model impairs memory function and neurogenesis in C57BL/6 mice. Brain Behav Immun.

[132] Bicknell B, Liebert A, Johnstone D, Kiat H (2019). Photobiomodulation of the microbiome: implications for metabolic and inflammatory diseases. Lasers Med Sci.

[133] Bicknell B, Laakso EL, Liebert A, Kiat H (2022). Modifying the microbiome as a potential mechanism of photobiomodulation: a case report. Photobiomodul Photomed Laser Surg.

[134] Monsonego A, Nemirovsky A, Harpaz I (2013). CD4 T cells in immunity and immunotherapy of Alzheimer’s disease. Immunology.

[135] Beers DR, Henkel JS, Zhao W, Wang J, Appel SH (2008). CD4+ T cells support glial neuroprotection, slow disease progression, and modify glial morphology in an animal model of inherited ALS. Proc Natl Acad Sci U S A.

[136] Mittal K, Eremenko E, Berner O, Elyahu Y, Strominger I, Apelblat D (2019). CD4 T cells induce a subset of MHCII-expressing microglia that attenuates Alzheimer pathology. iScience.

[137] Dansokho C, Ait Ahmed D, Aid S, Toly-Ndour C, Chaigneau T, Calle V (2016). Regulatory T cells delay disease progression in Alzheimer-like pathology. Brain.

[138] Bostrom P, Wu J, Jedrychowski MP, Korde A, Ye L, Lo JC (2012). A PGC1-alpha-dependent myokine that drives brown-fat-like development of white fat and thermogenesis. Nature.

[139] Wrann CD, White JP, Salogiannnis J, Laznik-Bogoslavski D, Wu J, Ma D (2013). Exercise induces hippocampal BDNF through a PGC-1alpha/FNDC5 pathway. Cell Metab.

[140] Li DJ, Li YH, Yuan HB, Qu LF, Wang P (2017). The novel exercise-induced hormone irisin protects against neuronal injury via activation of the Akt and ERK1/2 signaling pathways and contributes to the neuroprotection of physical exercise in cerebral ischemia. Metabolism.

[141] Lourenco MV, Frozza RL, de Freitas GB, Zhang H, Kincheski GC, Ribeiro FC (2019). Exercise-linked FNDC5/irisin rescues synaptic plasticity and memory defects in Alzheimer’s models. Nat Med.

[142] Islam MR, Valaris S, Young MF, Haley EB, Luo R, Bond SF (2021). Exercise hormone irisin is a critical regulator of cognitive function. Nat Metab.

[143] Arikan S, Alaca N, Ozbeyli D, Elmas MA, Arbak S, Suyen G (2022). Effects of moderate aerobic exercise, low-level laser therapy, or their combination on muscles pathology, oxidative stress and irisin levels in the mdx mouse model of Duchenne muscular dystrophy. Lasers Med Sci.

[144] Covatti C, Mizobuti DS, Rocha GLD, da Silva HNM, de Lourenco CC, Pertille A (2023). Low-level photobiomodulation therapy modulates H(2)O(2) production, TRPC-6, and PGC-1alpha levels in the dystrophic muscle. Photobiomodul Photomed Laser Surg.

[145] Li Y, Dong Y, Yang L, Tucker L, Zong X, Brann D (2021). Photobiomodulation prevents PTSD-like memory impairments in rats. Mol Psychiatry.

[146] Lapchak PA, Boitano PD (2016). Transcranial near-infrared laser therapy for stroke: how to recover from futility in the NEST-3 clinical trial. Acta Neurochir Suppl.

[147] Heiskanen V, Hamblin MR (2018). Photobiomodulation: lasers vs. light emitting diodes?. Photochem Photobiol Sci.

[148] Litscher G, Min L, Passegger CA, Litscher D, Li M, Wang M, et al. Transcranial yellow, red, and infrared laser and LED stimulation: changes of vascular parameters in a chick embryo model. Integr Med Int. 2015;2:80–9.

[149] Litscher D, Litscher GJ (2014). Laser therapy and stroke—quantification of methodological requirements in consideration of yellow laser. Akupunktur Aurikulomed.

[150] Jagdeo JR, Adams LE, Brody NI, Siegel DM (2012). Transcranial red and near infrared light transmission in a cadaveric model. PLoS ONE.

[151] Tedford CE, DeLapp S, Jacques S, Anders J (2015). Quantitative analysis of transcranial and intraparenchymal light penetration in human cadaver brain tissue. Lasers Surg Med.

[152] Yaroslavsky AN, Schulze PC, Yaroslavsky IV, Schober R, Ulrich F, Schwarzmaier HJ (2002). Optical properties of selected native and coagulated human brain tissues in vitro in the visible and near infrared spectral range. Phys Med Biol.

[153] Henderson TA, Morries LD (2015). Near-infrared photonic energy penetration: can infrared phototherapy effectively reach the human brain?. Neuropsychiatr Dis Treat.

[154] El Khoury H, Mitrofanis J, Henderson LA (2019). Exploring the effects of near infrared light on resting and evoked brain activity in humans using magnetic resonance imaging. Neuroscience.

[155] Zomorrodi R, Loheswaran G, Pushparaj A, Lim L (2019). Pulsed near infrared transcranial and intranasal photobiomodulation significantly modulates neural oscillations: a pilot exploratory study. Sci Rep.

[156] Zivin JA, Albers GW, Bornstein N, Chippendale T, Dahlof B, Devlin T (2009). Effectiveness and safety of transcranial laser therapy for acute ischemic stroke. Stroke.

[157] Figueiro Longo MG, Tan CO, Chan ST, Welt J, Avesta A, Ratai E (2020). Effect of transcranial low-level light therapy vs sham therapy among patients with moderate traumatic brain injury: a randomized clinical trial. JAMA Netw Open.

[158] Salehpour F, Khademi M, Hamblin MR (2021). Photobiomodulation therapy for dementia: a systematic review of pre-clinical and clinical studies. J Alzheimers Dis.

[159] Pan WT, Liu PM, Ma D, Yang JJ (2023). Advances in photobiomodulation for cognitive improvement by near-infrared derived multiple strategies. J Transl Med.

[160] Liu D, Li W, Jiang X, Bai S, Liu J, Liu X (2019). Using near-infrared enhanced thermozyme and scFv dual-conjugated Au nanorods for detection and targeted photothermal treatment of Alzheimer’s disease. Theranostics.

[161] Ge K, Mu Y, Liu M, Bai Z, Liu Z, Geng D (2022). Gold nanorods with spatial separation of CeO(2) deposition for plasmonic-enhanced antioxidant stress and photothermal therapy of Alzheimer’s disease. ACS Appl Mater Interfaces.

[162] Ailioaie LM, Ailioaie C, Litscher G (2023). Photobiomodulation in Alzheimer’s Disease-A complementary method to state-of-the-art pharmaceutical formulations and nanomedicine?. Pharmaceutics.

[163] Meynaghizadeh-Zargar R, Salehpour F, Hamblin MR, Mahmoudi J, Sadigh-Eteghad S (2019). Potential application of upconverting nanoparticles for brain photobiomodulation. Photobiomodul Photomed Laser Surg.

[164] Huang YY, Chen AC, Carroll JD, Hamblin MR (2009). Biphasic dose response in low level light therapy. Dose Response.

[165] Huang YY, Sharma SK, Carroll J, Hamblin MR (2011). Biphasic dose response in low level light therapy - an update. Dose Response.

[166] Sharma SK, Kharkwal GB, Sajo M, Huang YY, De Taboada L, McCarthy T (2011). Dose response effects of 810 nm laser light on mouse primary cortical neurons. Lasers Surg Med.

[167] Rojas JC, Bruchey AK, Gonzalez-Lima F (2012). Low-level light therapy improves cortical metabolic capacity and memory retention. J Alzheimers Dis.

[168] Beckman D, Chakrabarty P, Ott S, Dao A, Zhou E, Janssen WG (2021). A novel tau-based rhesus monkey model of Alzheimer’s pathogenesis. Alzheimers Dement.

[169] Tu Z, Yan S, Han B, Li C, Liang W, Lin Y (2023). Tauopathy promotes spinal cord-dependent production of toxic amyloid-beta in transgenic monkeys. Signal Transduct Target Ther.

[170] Salehpour F, Hamblin MR, DiDuro JO (2019). Rapid reversal of cognitive decline, olfactory dysfunction, and quality of life using multi-modality photobiomodulation therapy: case report. Photobiomodul Photomed Laser Surg.

[171] Maksimovich IV (2015). Dementia and cognitive impairment reduction after laser transcatheter treatment of Alzheimer’s disease. World J Neurosci.

[172] Sipion M, Ferreira FM, Scholler J, Brana C, Gora M, Kouvas G (2023). A randomized, blinded study of photobiomodulation in a mouse model of Alzheimer’s disease showed no preventive effect. Sci Rep.

[173] Kheradmand A, Donboli S, Tanjani PT, Farhadinasab A, Tabeie F, Qutbi M (2022). Therapeutic effects of low-level laser therapy on cognitive symptoms of patients with dementia: a double-blinded randomized clinical trial. Photobiomodul Photomed Laser Surg.

[174] Nagy EN, Ali AY, Behiry ME, Naguib MM, Elsayed MM (2021). Impact of combined photo-biomodulation and aerobic exercise on cognitive function and quality-of-life in elderly Alzheimer patients with anemia: a randomized clinical trial. Int J Gen Med.

[175] van Dyck CH, Swanson CJ, Aisen P, Bateman RJ, Chen C, Gee M (2023). Lecanemab in early Alzheimer’s disease. N Engl J Med.

[176] Mintun MA, Lo AC, Duggan Evans C, Wessels AM, Ardayfio PA, Andersen SW (2021). Donanemab in early Alzheimer’s disease. N Engl J Med.

[177] Kivipelto M, Mangialasche F, Ngandu T (2018). Lifestyle interventions to prevent cognitive impairment, dementia and Alzheimer disease. Nat Rev Neurol.

[178] Sindi S, Mangialasche F, Kivipelto M (2015). Advances in the prevention of Alzheimer’s disease. F1000Prime Rep.

[179] Leuzy A, Mattsson-Carlgren N, Palmqvist S, Janelidze S, Dage JL, Hansson O (2022). Blood-based biomarkers for Alzheimer’s disease. EMBO Mol Med.

[180] Ossenkoppele R, van der Kant R, Hansson O (2022). Tau biomarkers in Alzheimer’s disease: towards implementation in clinical practice and trials. Lancet Neurol.

[181] Olsson B, Lautner R, Andreasson U, Ohrfelt A, Portelius E, Bjerke M (2016). CSF and blood biomarkers for the diagnosis of Alzheimer’s disease: a systematic review and meta-analysis. Lancet Neurol.

[182] Wang X, Huang W, Su L, Xing Y, Jessen F, Sun Y (2020). Neuroimaging advances regarding subjective cognitive decline in preclinical Alzheimer’s disease. Mol Neurodegener.

